# Persistent Ambipolar Heptacenes and Their Redox Species

**DOI:** 10.1002/anie.202200918

**Published:** 2022-05-05

**Authors:** Nico Zeitter, Nikolai Hippchen, Steffen Maier, Frank Rominger, Andreas Dreuw, Jan Freudenberg, Uwe H. F. Bunz

**Affiliations:** ^1^ Organisch-Chemisches Institut Ruprecht-Karls-Universität Heidelberg Im Neuenheimer Feld 270 69120 Heidelberg Germany; ^2^ Interdisciplinary Center for Scientific Computing Ruprecht-Karls-Universität Heidelberg Im Neuenheimer Feld 205 69120 Heidelberg Germany; ^3^ Centre of Advanced Materials (CAM) Ruprecht-Karls-Universität Heidelberg Im Neuenheimer Feld 225 69120 Heidelberg Germany

**Keywords:** Acenes, Heptacene, Nonacene, Polycyclic Aromatic Hydrocarbons, Steric Shielding

## Abstract

Sixfold TIPS‐ethynylation combined with fourfold bromination of the armchair edges furnishes a long‐lived, soluble heptacene; π‐extension via Stille coupling accesses a persistent tetrabenzononacene. Both types of acenes were stabilized best by double TIPS‐ethynylation on every other benzene ring. Tetrabromoheptacene is an ambipolar transistor material (up to 0.036 cm^2^ V^−1^ s^−1^ n‐channel), which was corroborated by generation of its monoanion and monocation.

## Graphical abstract

Soluble heptacenes are stabilized via TIPS‐ethynylation and halogenation. TIPS‐ethynylation on every second ring is critical for longevity. The most stable derivative is characterized in thin‐film transistors and post‐functionalized via Stille coupling to a nonarene.

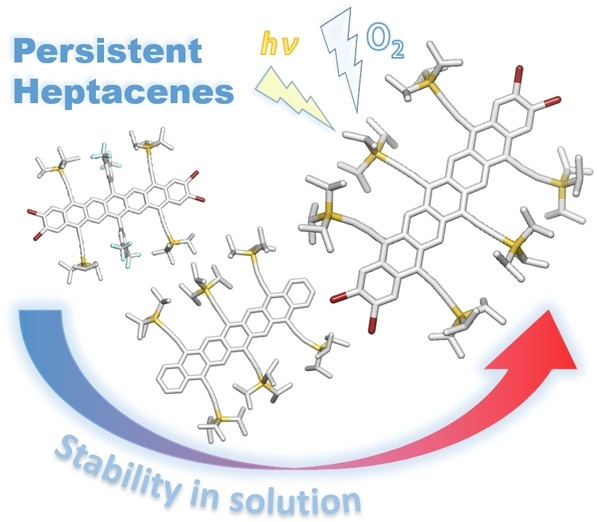

The synthesis of persistent yet soluble heptacenes is challenging.^[1]^ Their stabilization can be achieved through zig‐zag edge substitution (Figure 1),^[2]^ such as in **A**.^[2]^ Phenyl (**B**),^[3]^ and trifluoromethylphenyl substituents (**C**)^[4]^ improve stability but none survives more than a few hours under ambient conditions (solution), impeding processing and further functionalization.

**Figure 1:**
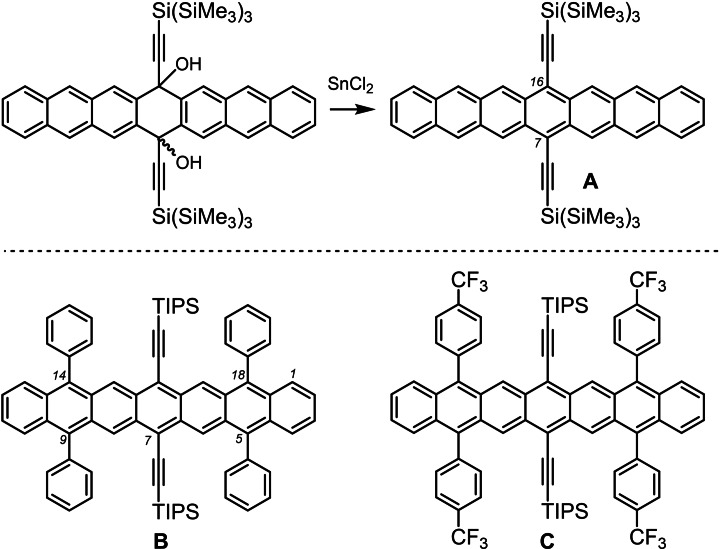
Previously reported, stabilized heptacenes **A**,^[2]^ **B**,^[3]^ and **C**.^[4]^

Transformations at the intact acene backbone are rare, even for pentacenes.^[5]^ Synthesis of higher acenes^[6]^ involves “protected” acenes (e.g. Figure 1, top), whose backbones, containing anthracene fragments, are aromatized in the final step to avoid handling of sensitive compounds.[^2^, ^4^, ^7^]

In this contribution, we present three long‐lived heptacenes **1** **a**–**c** with six strategically placed TIPS‐ethynyl groups. **1** **a** displays improved stability compared to **1** **b** substituted with two bis(trifluoromethyl)phenyl rings at its center. Bromination further increases their stability (cf. **1** **c**, Scheme 1). Persistability was demonstrated via UV/Vis measurements—we extended **1** **a** into a persistent nonacene, more robust than its arylated homologue.^[7b]^

**Scheme 1:**
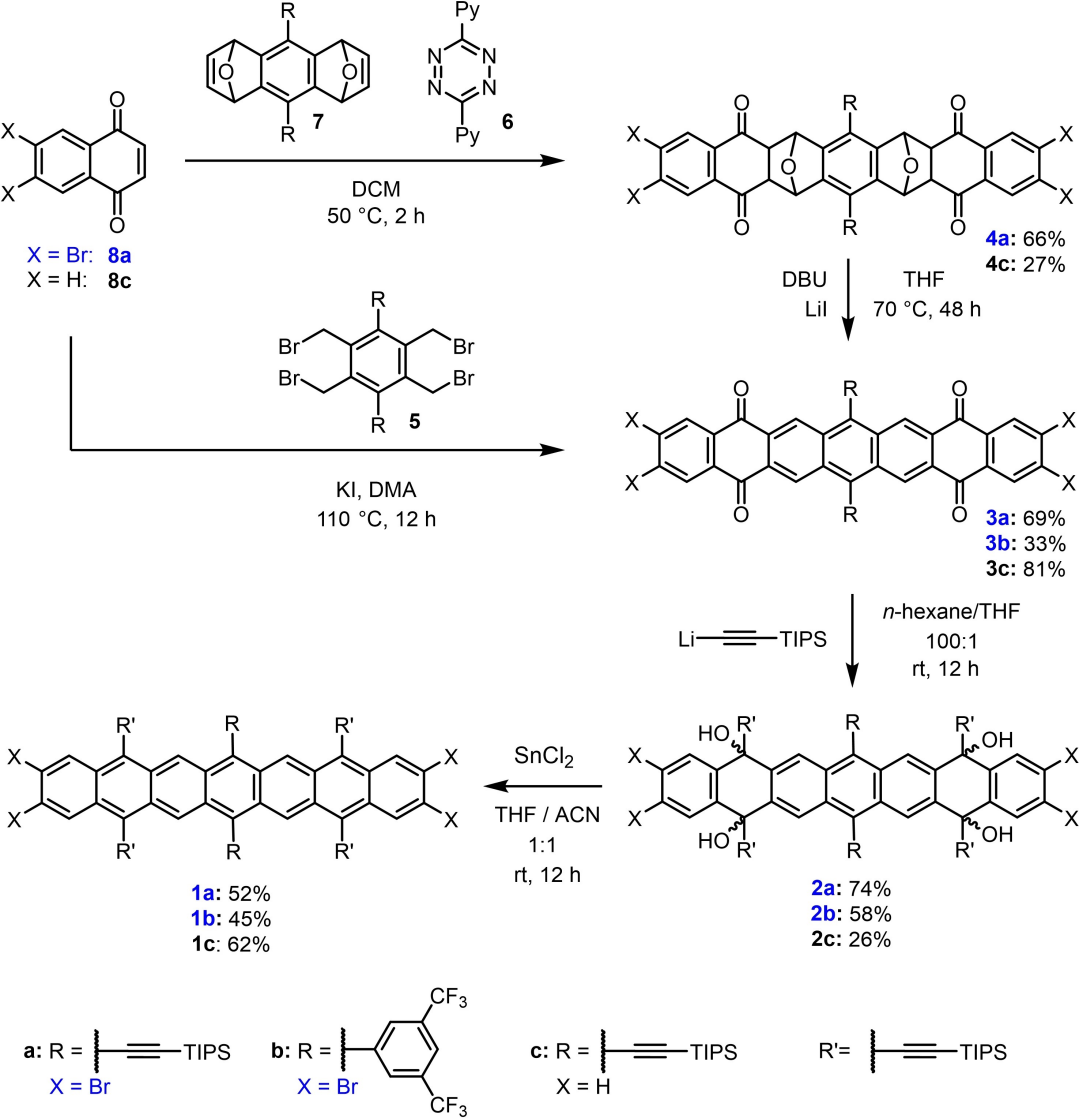
Synthesis of tetrabromoheptacenes **1** **a** and **1** **b** and non‐halogenated heptacene **1** **c**.

Naphthoquinones **8** **a** or **8** **c**,^[8]^ diepoxyanthracene **7** (Supporting Information)^[9]^ and **6** were reacted in a sequence of Diels–Alder (DA) and retro‐DA reactions to give **4** **a/c** as a mixture of diastereomers, which were deoxygenated into **3** **a/c** with DBU and LiI (Scheme 1). Alternatively, **8** **a** combined with **5** in a double Cava reaction[^7a^, ^7c^, ^9^] into **3** **b**. The quadruple ethynylation of **3** **a**–**c** with lithiated TIPS acetylene (100 equiv) gave a diastereomeric mixture of the intermediates **2** **a**–**c**; SnCl_2_ furnished **1** **a**–**c** almost quantitatively. **1** **a**–**c** precipitate as dark brown solids, moderately soluble in THF, toluene and DCM after column chromatography under air(!), and are stable for weeks as solids under N_2_.

**1** **a**–**c** (Figure 2, X‐ray crystal structures) pack in a herringbone motif. π–π interactions are absent due to the bulky side groups directed towards the π‐surface of the neighboring acenes. The aryl groups in **1** **b** are oriented perpendicularly (83°) to the central ring leading to only slight bending of its alkynes. In **1** **a** and **1** **c** (two independent molecules per unit cell for the latter), the outer alkynyl groups bend to escape steric strain. The acene backbones are deformed: in **1** **a** sigmoidally and in **1** **c** the central ring is twisted out of the acene plane.

**Figure 2:**
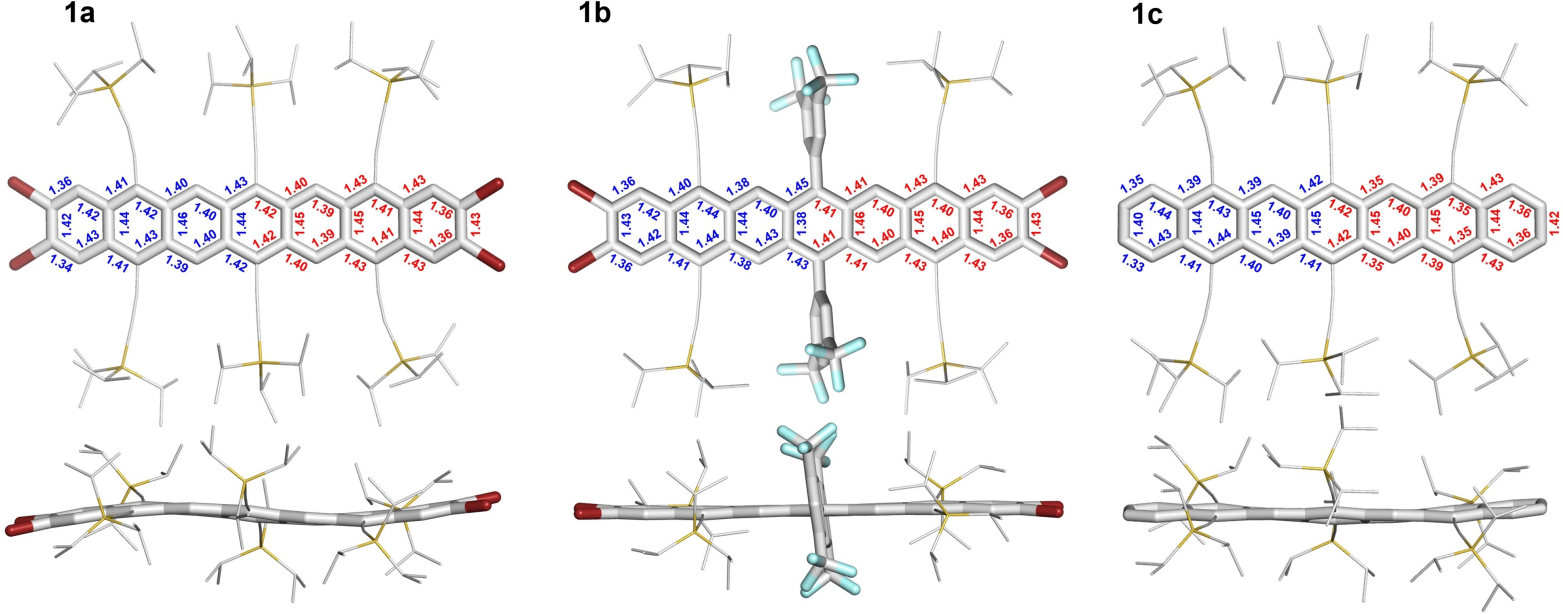
X‐ray single crystal structures, obtained from evaporation THF solutions under inert atmosphere, of **1** **a**–**c** (top and side view) including bond lengths (in Å) determined by crystal analysis (blue) vs. calculated bond lengths (red; DFT, B3LYP/def2‐TZVP). For the sake of clarity, all protons were omitted and TIPS‐ethynyl substituents were reduced in size. For **1** **c**, only one of the two independent molecules is depicted (see Supporting Information, Figure S32 for second molecule). For calculations, TIPS substituents were replaced with trimethylsilyl (TMS) groups.

**1** **a**–**c** absorbs with acene‐typical vibronically structured π‐bands in the (near‐)infrared (Figure 3). The shoulder near the absorption onset is typical of heptacenes[^3^, ^4^] and indicative of a doubly excited state of partially diradicaloid systems.^[10]^ *λ*_max_ of **1** **b** (870 nm, shoulder at 910 nm) is blue shifted in comparison to that of **1** **a** (908 nm, shoulder at 946 nm). Formal debromination to **1** **c** (888 nm, 927 nm shoulder) results in a hypsochromic shift. The optical band gaps amount to 1.25 eV for **1** **a**, 1.30 eV for **1** **b** and 1.27 eV for **1** **c** (Tables 1, 2) with electron affinities of **1** **a** (−4.18 eV), **1** **b** (−4.15 eV) and **1** **c** (−4.06 eV) according to cyclic voltammetry, promising n‐channel activity.

**Figure 3:**
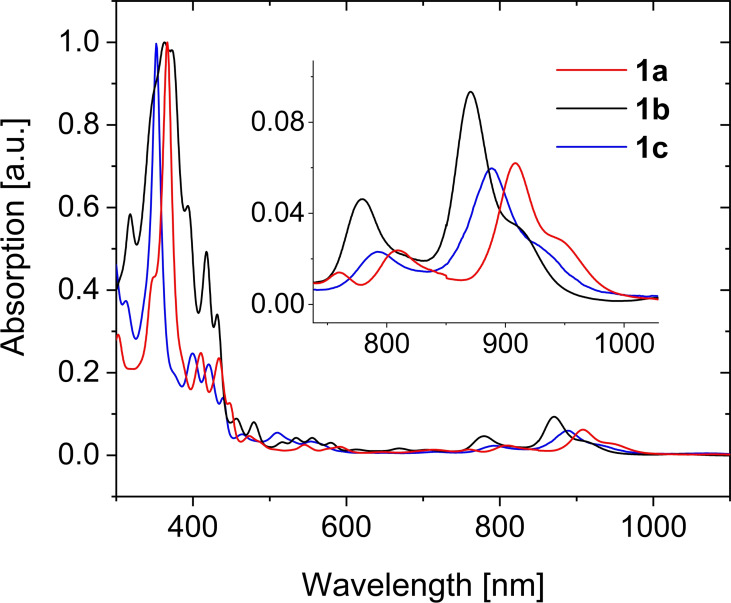
Normalized UV/Vis absorption spectra of **1** **a** (red), **1** **b** (black) and **1** **c** (blue) in toluene. Inset: enlargement of absorption maxima.

**Table 1:** Photophysical and calculated properties of **B**,^[3]^ **C**,^[4]^ **1** **a**, **1** **b**, **1** **c** and **9** **a**.

Compound	*λ*_onset_, [nm]^[a]^	*E*_gap, opt_ [eV]^[b]^	*E*_red1_ [eV]^[c]^	*E*_ox1_ [eV]^[c]^	*E*_gap, CV_ [eV]^[d]^	EA_CV_ [eV]^[e]^	IP_CV_ [eV]^[f]^	*E*_gap, DFT_ [eV]^[g]^
**B**	917	1.35	−1.13	0.25	1.38	−3.5	−4.8	–
**C**	917	1.35	−1.33	0.25	1.58	−3.61	−4.93	–
**1** **a**	991	1.25	−0.92	0.60	1.52	−4.18	−5.43	1.16
**1** **b**	951	1.30	−0.95	0.50	1.45	−4.15	−5.45	1.27
**1** **c**	980	1.27	−1.04	0.55	1.59	−4.06	−5.33	1.18
**9** **a**	1024	1.21	−1.06	0.46	1.52	−4.04	−5.25

[a] Onset of the lowest energy absorption maxima. [b] Optical gap calculated by λ_onset_. [c] First reduction and oxidation potentials measured by cyclic voltammetry (CV) in THF using Bu_4_NPF_6_ as electrolyte vs. Fc/Fc^+^ as internal standard (−5.1 eV)^[11]^ at 0.2 Vs^−1^. [d] Estimated by *E*_gap, CV_=*E*_ox1_−*E*_red1_. [e] Electron affinities estimated from first reduction potentials (EA_CV_=−5.10 eV−*E*_red1_). [f] Estimated using the approximation: IP_CV_=EA_CV_−*E*_gap, opt_. [g] *E*_gap, DFT_ obtained from DFT calculations (Gaussian 16,^[12]^ B3LYP/def2‐TZVP)_,_TMS groups were used to approximate TIPS substituents.

**Table 2:** Absorption maxima and stabilities of **A**,^[2]^ **B**,^[3]^ **C**,^[4]^ **1** **a**, **1** **b**, **1** **c** and **9** **a**.

Compound	*λ*_max._ [nm] (toluene)^[a]^	Degradation time (toluene, ambient conditions)^[b]^	Degradation time (toluene, nitrogen atmosphere)^[c]^
**A**	851	a few hours^[d]^	n.a.
**B**	863	41 h	n.a.
**C**	870	47 h	66 h
**1** **a**	908, 945	219 h^[e]^	614 h^[e]^
**1** **b**	870, 910	105 h^[e]^	206 h^[e]^
**1** **c**	888, 927	76 h^[e]^	308 h^[e]^
**9** **a**	995	35 h^[e]^	n.a.

[a] Lowest energy absorption maxima with their shoulders. [b] Time until full decomposition in toluene under ambient conditions. [c] Time until full decomposition in toluene under nitrogen atmosphere. [d] In DCM. [e] Extrapolated from absorbance decay (see Supporting Information). n.a.=Data not available. Note that for consistency reasons with values provided in literature, we provide degradation times rather than half‐life times.

Compared to **C** and **B** the electron affinity of **1** **a**–**c** is increased due to the bromine (Table 1), silylethynyl and *m*‐bis(trifluoromethyl)phenyl groups; yet **1** **a**–**c** were oxidized (0.60 V reversible, **1** **a**; 0.50 V, onset, irreversible, **1** **b**; 0.55 V reversible, **1** **c**). DFT gives LUMO levels (B3LYP, def2‐SVP) in accord with experimental electron affinities (LUMO_DFT_=−3.76 eV, −3.90 eV, −3.49 eV for **1** **a**–**c**). HOMO levels of **1** **a**, **b** are lowered (**1** **a**: −4.92 eV, **1** **b**: −5.18 eV) compared to de‐bromo **1c** (HOMO: −4.68 eV). TIPS groups were replaced by TMS substituents to simplify the calculations.

Silylethynylated acenes form peroxides, or dimerize in [4+4] or [2+4] cycloadditions,^[13]^ suppressed by bulky substituents; oxidation is retarded by reducing the ionization potential—criteria fulfilled in **1** **a**, **b** (and, to some extent, **1** **c**), particularly when compared to **A**–**C**. Stabilities of **1** **a**–**c** were investigated in dilute toluene solution (*c*≈10^−6^ M) under nitrogen as well as ambient conditions (Supporting Information, Section 2.3, Figures S26–S29).

The p‐bands of **1** **a** disappear after 614 h (≈25 d) under N_2_ and 219 h (≈9 d) under ambient conditions. **1** **b** and **1** **c** are less stable (**1** **b**: N_2_: ≈8 d, ambient: ≈5 d; **1** **c**: N_2_ ≈13 d, ambient: ≈3 d) but still persistent. It was observed that TIPS‐ethynyl substituents alone are better than a combination of aryl and TIPS‐ethynyl groups while stabilizing 5,7,9,14,16,18‐substituted heptacenes. **1** **a**–**c** are more stable (Table 2) than the current record holder **C**.^[4]^

Absorption bands ascribed to tetracene units (500–600 nm) appear during photodegradation.^[5d]^ ^1^H‐NMR and MALDI mass spectra (*m*/*z* 1792 [*M*+O]^+^; *m*/*z* 1808 [*M*+O2]^+^) of **1a**’s orange degradation product (light, air, toluene) suggest the formation of an *endo*‐peroxide **1** **a‐O2** (Supporting Information, Scheme S34) at one of the inner, less shielded benzene rings.

**1** **a** and **1** **c** exhibit ambipolar transport in bottom gate/top contact field‐effect transistors (Figure 4; Supporting Information 2.7, Figure S39 and S40) with μ_n‐max_=3.6×10^−2^ cm^2^ V^−1^ s^−1^ and μ_p‐max_=0.98×10^−2^ cm^2^ V^−1^ s^−1^ for **1** **a** (**1** **c**: μ_n‐max_=7.7×10^−4^ cm^2^ V^−1^ s^−1^, μ_p‐max_=1.7×10^−3^ cm^2^ V^−1^ s^−1^). Grazing X‐ray diffraction (see Supporting Information Section 2.8, Figure S41) reveals that **1** **a**, **c** pack similar in thin films as in single crystals. **1** **a** and **1** **c** orient in an edge‐on fashion. In contrast to other trialkylsilylethynyl‐substituted acenes, the armchair edges *and not the trialkylsilyl groups* point towards the substrate. The perpendicular π‐planes, (**1** **a**: 88.4°, **1** **c**: 89.7°), facilitate charge carrier transport.^[14]^

**Figure 4:**
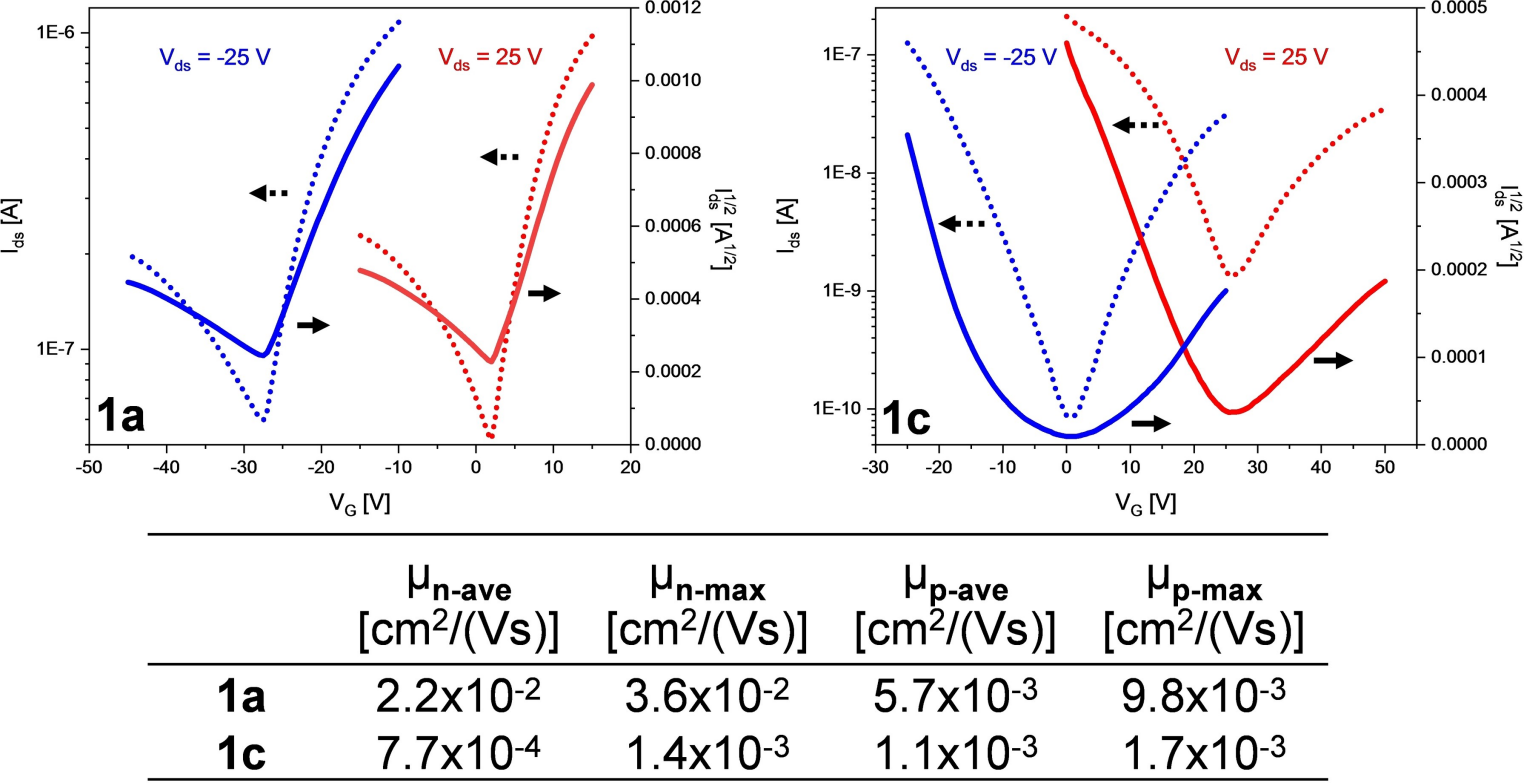
Top: Transfer characteristics of **1** **a** (left) and **1** **c** (right). Bottom: Charge transfer mobilities of **1** **a** and **1** **c**. At least six channels from two different substrates were measured to obtain averaged (av) mobility.

We oxidized **1** **a** with AgSbF_6_ and reduced **1** **a** with potassium anthracenide in the presence of 18‐crown‐6 (Scheme 2). Reddish‐purple solutions of **1** **a^+^** ⋅ and **1** **a** ^−.^ were EPR active with g_iso_=2.003 (Figure 5, left and middle). Both coupled with four I=1/2 nuclei—the charges reside in the center of the heptacene π‐systems as simulated spectra give α(^1^H, **1** **a^+^** ⋅)=7.51 MHz and α(^1^H, **1** **a** ^−.^)=6.76 MHz. As expected, absorption red shifts upon oxidation/reduction to 1453 nm (**1** **a** ^−.^) and 1584 nm (**1** **a^+^** ⋅), respectively (Supporting Information, Scheme S30). Both radical ions are persistent under ambient conditions with half‐lives of *t*_1/2_=8.7 h (anion) and 6.2 h (cation). A single crystal structure of **1** **a** ^−.^(Figure 5, right demonstrates that the potassium counterion is complexed and well separated from the aromatic species. The bond lengths of the aromatic backbone barely changed between the neutral and anionic form (See Supporting Information, Section 2.5, Figure S37).

**Scheme 2:**
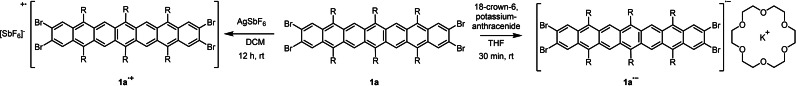
Oxidation and reduction of **1** **a** to its monocation **1** **a** ^.**+**^ and its monoanion **1** **a** ^.‐^.

**Figure 5:**
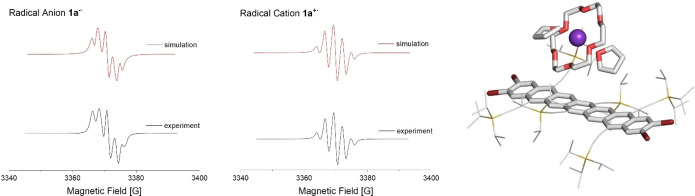
Left: Measured (THF, rt) and simulated EPR spectra of radical monoanion **1** **a** ^.−^ (top) and monocation **1** **a** ^.**+**^ (middle). Right: Single crystal structure of the mono‐reduced species of **1** **a**.

**1** **a** is sufficiently stable to react with **10** to give **9** **a** ^[16]^ (Scheme 3), stable for several weeks as a solid in the glovebox. **9** **a**, sparingly soluble in THF or DCM (≈1 mg mL^−1^), packs in a brickwall motif with π–π overlap of the triphenylene wings and a layer distance of 348 pm (Scheme 3 and Supporting Information, Section 2.5 Figure S34). **9** **a** (*t*_1/2_=8 h, toluene, *λ*_max_=995 nm) is more stable than **9** **b** (R′=3,5‐(CF_3_)_2_Ph, *t*_1/2_=30 min in hexanes, *λ*_max_=958 nm)^[7b]^ again highlighting the stabilizing power of amassed TIPS‐ethynyl groups for higher acenes.

**Scheme 3:**
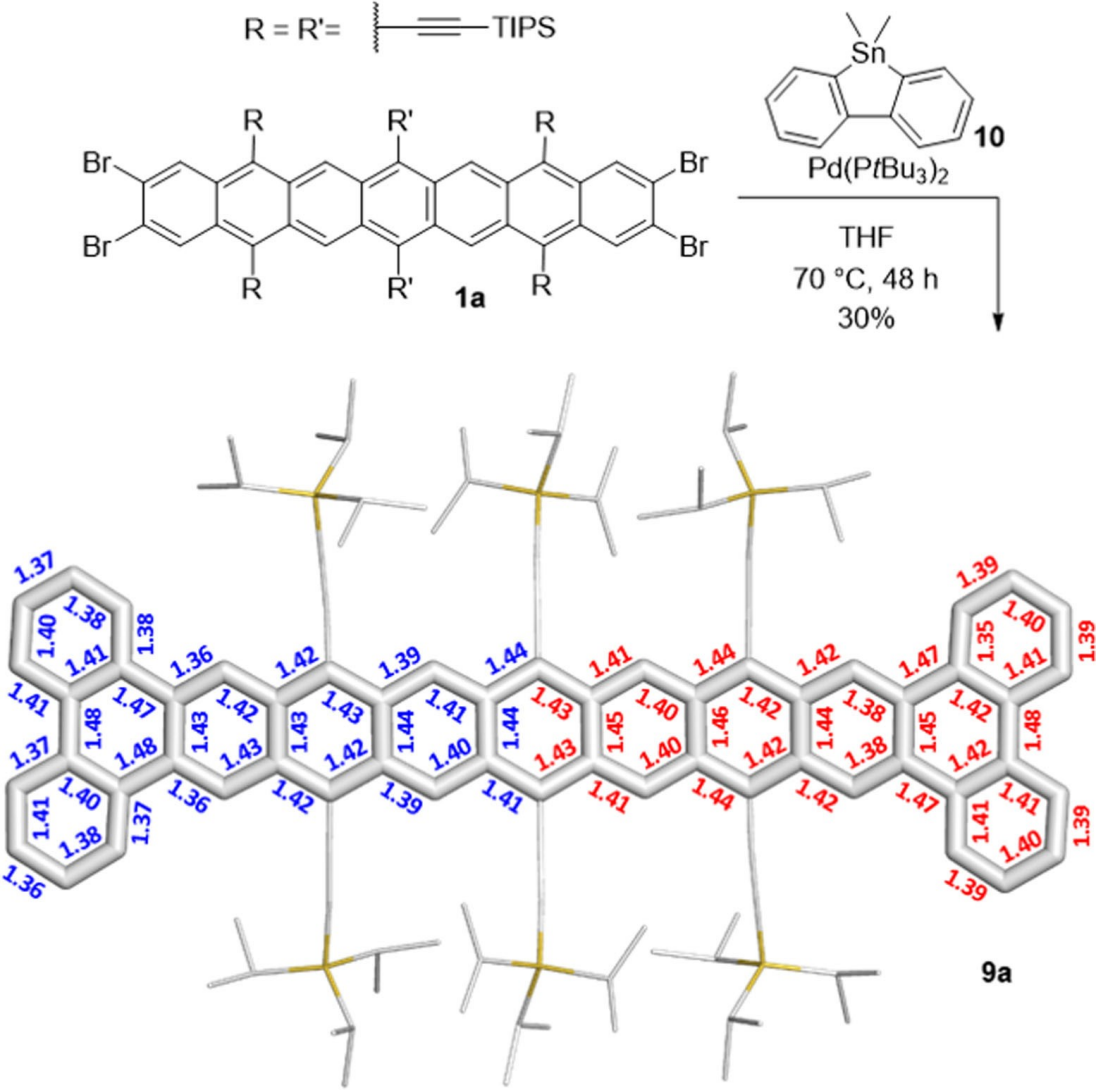
Synthesis of nonacene **9** **a** with its X‐ray single crystal structure (top view) including bond lengths (in Å) determined by crystal analysis (blue) vs. calculated bond lengths (red; Spartan’20 (1.0.0),^[15]^ DFT, B3LYP/6‐31G*). For the sake of clarity, all protons were omitted and TIPS‐ethynyl substituents were reduced in size.

It was possible to obtain a single crystal of **9** **a** directly from the reaction solution. However, it was not possible to isolate nonacene **9** **a** in bulk by chromatography or to characterize it via NMR spectroscopy. **9** **a** probably displays a triplet ground state and therefore NMR signals are not expected. It was characterized via EPR spectroscopy and a signal with g=1.9982 was observed (Supporting Information, Section 2.9, Figure S41).

To conclude, we prepared heptacene derivatives **1** **a**–**c**. Bromination lowers ionization potentials, retarding oxidation. All of the targets are stabilized by placing TIPS‐ethynyl groups on every second ring. **1** **a**, **c** show ambipolar charge transport in OFETs. The charge transporting species of **1** **a** were studied in form of its radical ions. Postfunctionalization furnishes the persistent nonacene **9** **a**. **1** **a** is robust, soluble and a starting material for large acenes—hopefully beyond nonacenes.

## Crystallographic data

Deposition Numbers 2141664 (for **1a**), 2141665 (for **1b**), 2141666 (for **1c**), 2141667 (for **9a**), and 2141668 (for **1** **a** ^.−^) contain the supplementary crystallographic data for this paper. These data are provided free of charge by the joint Cambridge Crystallographic Data Centre and Fachinformationszentrum Karlsruhe Access Structures service.

## Conflict of interest

The authors declare no conflict of interest.

## Supporting information

As a service to our authors and readers, this journal provides supporting information supplied by the authors. Such materials are peer reviewed and may be re‐organized for online delivery, but are not copy‐edited or typeset. Technical support issues arising from supporting information (other than missing files) should be addressed to the authors.

Supporting Information

Supporting Information

## Data Availability

Data related to this article are available through heiDATA, the institutional research data repository of Heidelberg University, under https://doi.org/10.11588/data/CVD7WF.
