# Supplementary material for: Persistent Ambipolar Heptacenes and Their Redox Species
Source: Angew Chem Int Ed Engl. 2022 May 5;61(26):e202200918. doi: 10.1002/anie.202200918 (PMC9324111; doi:10.1002/anie.202200918)
Supplement: Supplementary file 1 — Supporting Information [file ANIE-61-0-s002.pdf]

Angewandte Chemie
Eine Zeitschrift der Gesellschaft Deutscher Chemiker

Supporting Information

Persistent Ambipolar Heptacenes and Their Redox Species

N. Zeitter, N. Hippchen, S. Maier, F. Rominger, A. Dreuw, J. Freudenberg, U. H. F. Bunz**

Table of Contents

1 Experimental Procedures 2
1.1 Materials and Methods 2
1.2 Synthesis 3
2 Characterization 3
2.1 NMR Spectroscopy 6
2.2 IR Spectroscopy 13
2.3 UV/vis Spectroscopy 19
2.4 Cyclic Voltammetry 20
2.5 Crystallographic Data 22
3 References 32
4 Author Contributions 32

1 Experimental Procedures

1.1 Materials and Methods

Column chromatography was performed using silica gel (SiO_2, pore size 60 Å, particle size 40-63 µm) manufactured by *SIGMA ALDRICH*. Melting points were determined in open glass capillaries on a Melting Point Apparatus MEL-TEMP (Electrothermal, Rochford, UK) and are uncorrected. X-ray single-crystal structure analyses of **1a**, **1b**, **1c**, and **9a** were measured on a STOE Stadivari CCD area detector diffractometer. Diffraction intensities were corrected for Lorentz and polarization effects. An empirical scaling and absorption correction was applied using X-Area LANA 1.70.0.0 based on the Laue symmetry of reciprocal space, structures were solved with SHELXT-2014[1] and refined against F^2 with a Full-matrix least-squares algorithm using the SHELXL-2018/3 (Sheldrick, 2018) software.[2] X-ray single-crystal structure analyses of **1a**$^{\cdot-}$ were measured on a Bruker APEX-II Quazar area detector. An empirical scaling and absorption correction was applied using SADABS based on the Laue symmetry of the reciprocal space, structure solved with SHELXT-2018/2 (Sheldrick 2015) and refined against F^2 with a Full-matrix least-squares algorithm using the SHELXL-2018/3 (Sheldrick, 2018) software.[2] NMR spectra were recorded on Bruker Avance III spectrometers using the specified frequency. Chemical shifts (δ) are given in parts per million (ppm) relative to internal solvent signals.[3] Signal multiplicities are described by the following abbreviations: s = singlet, d = doublet, dd = doublet of doublets, m = multiplet, bs = broad signal. High-resolution mass spectra (HRMS) were obtained by (matrix-assisted) laser desorption/ionization (LDI/MALDI) using trans-2-[3-(4-*tert*-butylphenyl)-2-methyl-2-propenylidene]malononitrile (DCTB) as matrix, electrospray ionisation (ESI) or direct analysis in real time (DART) experiments. IR spectra of the powdery analytes were recorded on a Jasco FT/IR-4100 spectrometer. Cyclovoltammographic (CV) measurements were performed on a VersaSTAT 3 potentiostat by Princeton Applied Research. UV-vis spectra were recorded on a *JASCO UV-VIS V-660* spectrometer using *HELLMA ANALYTICS* precision cells (10 mm, type 111-QS). All calculations were performed using Gaussian16. TMS groups were used instead of TIPS groups to simplify calculations. First, the gas-phase ground-state equilibrium geometry of the molecules was optimized at the B3LYP/def2-SVP level of theory. Afterwards, the recieved geometries were refined using the B3LYP/def2-TZVP level of theory. FMO calculations were performed starting from the optimized geometries on the B3LYP/def2-TZVP level of theory.[4] The geometry optimization calculation of **9a** was performed using Spartan'20, Version 1.0.0. TMS groups were used instead of TIPS groups to simplify calculations. The gas-phase ground-state equilibrium geometry of the molecule was optimized at the B3LYP/6-31G* level of theory.[5] 2',3',5',6'-Tetrakis(bromomethyl)-3,3'',5,5''-tetrakis(trifluoromethyl)-1,1':4',1''-terphenyl (**5**),[6] 9,10-bis((triisopropylsilyl)ethynyl)-1,4,5,8-tetrahydro-1,4:5,8-diepoxyanthracene (**7**)[7] and 6,7-dibromonaphthalene-1,4-dione (**8a**)[8] were synthesized according to literature procedures.

1.2 Synthesis

GPS1: The corresponding silyl acetylene (100 eq.) was dissolved in dry *n*-hexane under argon atmosphere. *n*-BuLi (95.0 eq., 2.50 M in *n*-hexane) was added dropwise at r.t. and the mixture was stirred for 30 min. The respective acenequinone (1.00 eq.) and small volumes of dry THF were added. The suspension was stirred for 12 h at room temperature (rt). The reaction was quenched with saturated aqueous ammonium chloride solution and extracted with DCM. The combined organic layers were dried over anhydrous magnesium sulfate and the solvent was removed under reduced pressure.

GPS2: The corresponding acenetetraol (1.00 eq.) was dissolved in a mixture of acetonitrile / THF (1:1) under argon atmosphere. Anhydrous $SnCl_2$ (10.0 eq.) was added, and the reaction mixture was stirred at r.t. overnight. The resulting mixture was filtered, and the precipitate was washed with acetonitrile until the filtrate was clear.

2,3,11,12-Tetrabromo-7,16-bis((triisopropylsilyl)ethynyl)-5a,6,8,8a,14a,15,17,17a-octahydro-6,17:8,15-diepoxyheptacene-5,9,14,18-tetraone (4a) mixture of geometric isomers

7 (500 mg, 876 µmol, 1.00 eq.), **8a** (830 mg, 2.63 mmol, 3.00 eq.) and **6** (414 mg, 1.74 mmol, 2.00 eq.) were dissolved in dry DCM (100 mL) under argon atmosphere and the solution was stirred at 50 °C for 4 h. Subsequently, the solvent was removed under reduced pressure and the crude product was purified by flash column chromatography (SiO_2, PE:EE 20:1) to yield an isomeric mixture of compound **4a** as a brown solid (670 mg, 582 µmol, 66%). The isomeric products were carried directly into the next step without further purification. Nevertheless, it was possible to separate one diastereomer from the isomeric mixture by further purification by flash column chromatography (SiO_2, PE:EE 20:1). ^{1}H- and ^{13}C-NMR. were measured with isomerically pure samples of compound **4a**. R_f (PE:EE; 4:1) = 0.60. Mp: = 305 °C. ^{1}H NMR (301 MHz, $CDCl_3$): δ [ppm]: 7.80 (s, 4H), 5.63 (dd, *J* = 3.5 Hz, 1.9 Hz, 4H), 3.69 (dd, *J* = 3.5 Hz, 1.9 Hz, 4H), 1.30 (s, 42H). ^{13}C{^{1}H} NMR (151 MHz, $CDCl_3$): δ [ppm]: 190.4, 143.9, 133.4, 132.3, 131.1, 111.2, 102.8, 82.2, 49.7, 18.9, 11.4. IR (ATR): $\tilde{\nu}$ [cm^{-1}]: 2938, 2861, 2358, 2339, 2159, 1686, 1570, 1459, 1300, 1259, 997, 942, 883, 856, 796, 666, 568, 504, 410.

7,16-Bis((triisopropylsilyl)ethynyl)-5a,6,8,8a,14a,15,17,17a-octahydro-6,17:8,15-diepoxyheptacene-5,9,14,18-tetraone (4c) mixture of geometric isomers

7 (1.00 g, 1.75 mmol, 1.00 eq.), **8c** (692 mg, 4.38 mmol, 2.50 eq.), and **6** (827 mg, 3.50 mmol, 2.00 eq.) were dissolved in dry DCM (100 mL) under argon atmosphere and the solution was stirred at 50 °C for 4 h. Subsequently, the solvent was removed under reduced pressure and the crude product was purified by flash column chromatography (SiO_2, PE:EE 20:1) to yield an isomeric mixture of compound **4c** as a brown solid (401 mg, 478 µmol, 27%). The isomeric products were carried directly into the next step without further purification. Nevertheless, it was possible to separate one diastereomer from the isomeric mixture by further purification by flash column chromatography (SiO_2, PE:EE 20:1). ^{1}H- and ^{13}C-NMR. were measured with isomerically pure samples of compound **4c**. R_f (PE:EE; 4:1) = 0.55. Mp: > 350 °C. ^{1}H NMR (301 MHz, $CDCl_3$): δ [ppm]: 7.59 - 7.56 (m 4H), 7.45 - 7.42 (m 4H), 5.55 - 5.54 (m 4H), 3.65 - 3.63 (m 4H), 1.29 (m 42H).^{13}C{^{1}H} NMR (151 MHz, $CDCl_3$): δ [ppm]: 192.2, 143.5, 134.2, 134.0, 125.9, 110.6, 101.3, 98.0, 82.1, 49.4, 18.8, 11.4. IR (ATR): $\tilde{\nu}$ [cm^{-1}]: 3062.89, 2929.34, 2864.74, 2361.41, 2333.93, 1683.07, 1584.24, 1569.77, 1413.08, 987.85, 872.14, 777.17, 759.81, 747.28, 726.06, 619.03, 587.21, 405.94.

2,3,11,12-Tetrabromo-7,16-bis((triisopropylsilyl)ethynyl)heptacene-5,9,14,18-tetraone (3a)

4a (500 mg, 434 µmol, 1.00 eq.), diazabicycloundecene (1.33 g, 1.30 mL, 8.69 mmol, 20.0 eq.) and lithium iodide (116 mg, 869 µmol, 2.00 eq.) were dissolved in dry THF (150 mL) under argon atmosphere and the mixture was refluxed overnight. After cooling to rt the reaction mixture the precipitate was collected by filtration and the orange brown residue was washed with THF (100 mL), water (50.0 mL) and methanol (50.0 mL) to yield an orange solid as the pure product (333 mg, 299 µmol, 69 %). **3a** was insoluble in common organic solvents impeding characterization via NMR spectroscopy. Mp: > 350 °C. HRMS (MALDI+, DCTB): m/z calcd. for $C_{52}H_{50}Si_2{}^{79}Br_2{}^{81}Br_2O_4$: [M$^+$] 1113.9946, found: 1113.9954, correct isotope distribution. IR (ATR): $\tilde{\nu}$ [cm^{-1}]: 3077, 2939, 2857, 2359, 1682, 1568, 1446, 1370, 1256, 995, 882, 732, 672.

7,16-Bis(3,5-bis(trifluoromethyl)phenyl)-2,3,11,12-tetrabromo-heptacene-5,9,14,18-tetraone (3b)

8 (451 mg, 1.43 mmol, 2.50 eq.) and **5** (500 mg, 572 µmol, 1.00 eq.) were dissolved in degassed *N,N*-dimethylacetamide (40 mL) under argon atmosphere. The mixture was heated to 110 °C and potassium iodide (1.42 g, 8.58 mmol, 15.0 eq.) was added. The reaction mixture was stirred for 12 h at 110 °C. During the reaction, a yellow precipitate formed. The hot reaction mixture was poured into water (500 mL), the precipitate was collected by filtration and washed with acetone and THF until the filtrate was clear. The insoluble brown precipitate was dried under reduced pressure to yield **3b** (220 mg, 186 µmol, 33 %). NMR characterization was unsuccessful due to the insolubility of the product. Mp: > 350 °C. HRMS (MALDI+, DCTB): m/z calcd. for $C_{46}H_{14}Br_4F_{12}O_4$: [M$^+$] 1177.7399 found: 1177.7391, correct isotope distribution. IR (ATR): $\tilde{\nu}$ [cm^{-1}]: 3083.62, 2360.44, 2341.16, 1686.44, 1570.74, 1278.57, 1186.01, 1127.19, 997.017, 920.843, 736.674, 710.64, 679.785, 502.366, 468.617.

7,16-Bis((triisopropylsilyl)ethynyl)heptacene-5,9,14,18-tetraone (3c)

Compound **4c** (261 mg, 312 µmol, 1.00 eq.), diazabicycloundecene (951 mg, 0.93 mL, 6.25 mmol, 20.0 eq.) and lithium iodide (83.65 mg, 625 µmol, 2.00 eq.) were dissolved in dry THF (75 mL) under argon atmosphere and the mixture was refluxed overnight. After cooling to rt the precipitate was collected via filtration and the orange brown residue was washed with THF (100 mL), water (50.0 mL) and methanol (50.0 mL) to yield an orange solid as the pure product **3c** (202 mg, 253 µmol, 81%).**3c** was insoluble in common organic solvents impeding characterization via NMR spectroscopy. Mp: > 350 °C. HRMS (MALDI+, DCTB): m/z calcd. for $C_{52}H_{54}Si_2O_4$: [M$^+$] 798.3555, found: 798.3556, correct isotope distribution. IR (ATR): $\tilde{\nu}$ [cm^{-1}]: 2938.5, 2863.77, 1682.11, 1590.99, 1443.94, 1270.38, 986.41, 896.25, 880.34, 803.20, 743.90, 713.53, 677.37, 661.46, 651.82, 627.71, 622.89.

2,3,11,12-Tetrabromo-5,7,9,14,16,18-hexakis((triisopropylsilyl) ethynyl)-5,9,14,18-tetrahydroheptacene-5,9,14,18-tetraol (2a), mixture of geometric isomers

GPS1 was performed with (triisopropylsilyl)acetylene (2.13 g, 2.62 mL, 11.7 mmol, 100 eq.) and heptacenediquinone **3a** (130 mg, 116 µmol, 1.00 eq.) in a mixture of dry *n*-hexane (20.0 mL) and dry THF (1.00 mL). The crude product was purified by flash column chromatography (SiO_2, PE:DCM 10:1) to yield an isomeric mixture of **2a** as a yellow solid (160 mg, 86.8 µmol, 74%). It was possible to separate one diastereomer from the isomeric mixture by further purification by flash column chromatography (SiO_2, PE:DCM 10:1). ^{1}H- and ^{13}C-NMR. were measured with isomerically pure samples of compound **2a**. R_f (PE:DCM; 3:1) = 0.65, 0.55. Mp: = 286 - 288 °C. ^{1}H NMR (600 MHz, $CDCl_3$) δ [ppm]: 9.34 (s, 4H), 8.44 (s, 4H), 3.08 (s, 4H), 1.29 (m, 42H), 0.99 (m, 42H).^{13}C{^{1}H} NMR (151 MHz, $CDCl_3$) δ [ppm] 138.6, 136.9, 132.9, 132.7, 126.6, 125.6, 119.8, 109.6, 107.8, 103.3, 90.4, 68.1, 19.2, 19.1, 18.7, 18.7, 11.9, 11.8, 11.4, 11.3. MS (MALDI+, DCTB): m/z calcd. for $C_{96}H_{139}Si_6Br_4O_4$: [M+H$^+$] 1845.3, found: 1845.1, correct isotope distribution. IR (ATR): $\tilde{\nu}$ [cm^{-1}]: 3460, 3281, 2938, 2858, 2362, 2128, 1619, 1456, 1373, 1210, 1042, 1000, 883, 666, 506. EA calcd. for $C_{96}H_{139}Si_6Br_4O_4$ C: 62.52% H: 7.54%, found: C: 62.64% H: 7.82%

7,16-Bis(3,5-bis(trifluoromethyl)phenyl)-2,3,11,12-tetrabromo-5,9,14,18-tetrakis((triisopropylsilyl)ethynyl)-5,9,14,18-tetrahydroheptacene-5,9,14,18-tetraol (2b), mixture of geometric isomers

GPS1 was performed with (triisopropylsilyl)acetylene (1.24 g, 1.52 mL, 6.79 mmol, 100 eq.) and heptacenediquinone **3b** (80.0 mg, 67.9 µmol, 1.00 eq.) in a mixture of dry *n*-hexane (15.0 mL) and dry THF (1.00 mL). The crude product was purified by flash column chromatography (SiO_2, PE:DCM 10:1) to yield an isomeric mixture of **2b** as a yellow solid (76.0 mg, 39.8 µmol, 58%). It was possible to separate one diastereomer from the isomeric mixture by further purification by flash column chromatography (SiO_2, PE:DCM 10:1). ^{1}H- and ^{13}C-NMR. were measured with isomerically pure samples of compound **2b**. R_f (PE:DCM; 3:1) = 0.60, 0.55. Mp: > 350 °C. ^{1}H NMR (600 MHz, CDCl3): δ [ppm] 8.33 (s, 4H), 8.22 (s, 4H), 8.17 (s, 2H), 8.03 (s, 2H), 7.97 (s, 2H), 3.09 (s, 4H), 0.96 (s, 84H). ^{13}C{^{1}H} NMR (151 MHz, $CDCl_3$): δ [ppm] 140.2, 138.6, 136.8, 135.8, 132.6, 132.1, 130.2, 125.8, 124.5, 124.2, 122.5, 107.9, 91.2, 68.3, 18.6, 11.1. MS (MALDI+, DCTB): m/z calcd. for $C_{90}H_{102}Br_4F_{12}O_4Si_4$: [M+H$^+$] 1908.746 found: 1908.802, correct isotope distribution. IR (ATR): $\tilde{\nu}$ [cm^{-1}]: 3422, 3269, 2942, 2859, 2357, 1684, 1456, 1335, 1267, 1137, 875, 670, 533.

2,3,11,12-Tetrabromo-5,7,9,14,16,18-hexakis((triisopropylsilyl) ethynyl)-5,9,14,18-tetrahydroheptacene-5,9,14,18-tetraol (2c), mixture of geometric isomers

GPS1 was performed with (triisopropylsilyl)acetylene (1.14 g, 1.40 mL, 6.26 mmol, 100 eq.) and heptacenediquinone **3c** (50.0 mg, 62.6 µmol, 1.00 eq.) in a mixture of dry *n*-hexane (7.00 mL) and dry THF (0.50 mL). The crude product was purified by flash column chromatography (SiO_2, PE:DCM 5:1) to yield an isomeric mixture of **2c** as a yellow solid (25.0 mg, 16.36 µmol, 26%). It was possible to separate one diastereomer from the isomeric mixture by further purification by flash column chromatography (SiO_2, PE:DCM 10:1). ^{1}H- and ^{13}C-NMR. were measured with isomerically pure samples of compound **2c**. R_f (PE:DCM; 1:1) = 0.55, 0.35. Mp: > 350 °C. ^{1}H NMR (600 MHz, $CDCl_3$) δ [ppm]: 9.39 (s, 4H), 8.19 – 8.17 (m, 4H), 7.49 – 7.48 (m. 4H) 3.01 (s, 4H), 1.31 (m, 42H), 0.97 (m, 84H).^{13}C{^{1}H} NMR (151 MHz, $CDCl_3$) δ [ppm] 137.6, 137.6, 132.9, 129.2, 127.4, 126.7, 119.6, 110.9, 107.3, 103.7, 89.0, 68.5, 19.1, 18.7, 11.8, 11.4. HRMS (MALDI+, DCTB): m/z calcd. for $C_{96}H_{143}Si_6O_4$: [M+H$^+$] 1527.9596, found: 1527.9599, correct isotope distribution. IR (ATR): $\tilde{\nu}$ [cm^{-1}]: 3525, 2942, 2864, 1463, 1367, 1042, 1019, 996, 899, 881, 774, 764, 744, 675, 664, 637, 582, 567, 562, 506, 467, 405.

((2,3,11,12-Tetrabromoheptacene-5,7,9,14,16,18-hexayl)hexakis(ethyne-2,1-diyl))hexakis(triisopropylsilane) (1a)

GPS2 was applied to **2a** (70.0 mg, 38.0 µmol, 1.00 eq.) to yield crude **1a** as a brown solid. Purification by flash column chromatography (SiO_2, PE:DCM 10:1) yielded pure **1a** as a brown solid (35.0 mg, 19.7 µmol, 52%). R_f(PE:DCM 10:1) = 0.8. Single crystalline specimen were obtained by evaporation of a concentrated dichloromethane solution. Mp: > 350 °C. ^{1}H NMR (500 MHz, $CDCl_3$) δ [ppm]: 9.83 (s, 4H), 8.92 (s, 4H), 1.30 (m, 84H), 1.20 (m, 42H). The compound was not soluble enough for ^{13}C[9] NMR analysis. IR (ATR): $\tilde{\nu}$ [cm^{-1}]: 2922, 2862, 2359, 2342, 1695, 1458, 1375, 1260, 1063, 1016.3, 882, 800, 728, 677, 570, 504. HRMS (MALDI+, DCTB): m/z calcd. for $C_{96}H_{138}Si_6{}^{79}Br_3{}^{81}Br_1$: [M$^+$] 1776.6122, found: 1776.6096, correct isotope distribution.

((7,16-Bis(3,5-bis(trifluoromethyl)phenyl)-2,3,11,12-tetrabromoheptacene-5,9,14,18-tetrayl)tetrakis(ethyne-2,1-diyl))tetrakis(triisopropylsilane) (1b)

GPS2 was applied to **2b** (30.0 g, 15.7 µmol, 1.00 eq.) was applied to **GP2** to furnish crude **1b** as a brown solid. Purification by flash column chromatography (SiO_2, PE:DCM 10:1) yielded pure **1b** as a brown solid (14.0 mg, 7.62 µmol, 45%).R_f(PE:DCM 10:1) = 0.4. Single crystal specimen were grown by subsequently layering MeOH on a concentrated THF solution of **1b**. Mp: > 350 °C. ^{1}H NMR (500 MHz, $CDCl_3$): δ [ppm]: 8.83 (s, 4H), 8.78(s, 4H), 8.21(s, 2H), 8.16(s, 4H), 1.13 (m, 42H). The compound was not soluble enough for ^{13}C[9] NMR analysis. IR (ATR): $\tilde{\nu}$ [cm^{-1}]: 2946, 2863, 2361, 2339, 2118, 1464, 1361, 1331, 1278, 1149, 879, 727, 662. HRMS (MALDI+, DCTB): m/z calcd. for $C_{90}H_{98}Si_4{}^{79}Br_3{}^{81}Br_1$: [M+H$^+$] 1839.3319, found: 1839.3352, correct isotope distribution.

(Heptacene-5,7,9,14,16,18-hexaylhexakis(ethyne-2,1-diyl))hexakis(triisopropylsilane) (1c)

GPS2 was applied to **2c** (51.0 mg, 33.4 µmol, 1.00 eq.) to furnish crude **1c** obtained as a dark brown solid. The crude product was purified by flash column chromatography (SiO_2, PE:DCM 10:1) to yield compound **1c** as a brown solid (30.0 mg, 20.5 µmol, 62 %). R_f(PE:DCM 10:1) = 0.9. Single crystal specimen were grown by subsequently layering MeOH on a concentrated THF solution of **1c**. Mp: > 350 °C. ^{1}H NMR (600 MHz, THF-d8) δ [ppm]: 9.97 (s, 4H), 8.61 - 8.60 (m, 4H), 7.52 - 7.51 (m. 4H), 1.49 - 1.39 (m, 18H), 1.34 - 1.33 (m, 72H) 1.24 - 1.23 (m, 36H). ^{13}C NMR (151 MHz, THF-d_8) δ [ppm]

134.56, 131.72, 130.86, 127.72, 127.66, 126.44, 119.13, 119.05, 112.64, 107.85, 107.14, 104.81, 18.74, 12.17.. IR (ATR): $\tilde{\nu}$ [cm^{-1}]: 2942, 2864, 2361, 2121, 1462, 1382, 1059, 1017, 996, 882, 757, 749, 673, 668, 653, 634, 506. HRMS (MALDI+, DCTB): m/z calcd. for $C_{96}H_{138}Si_6$: [M^+] 1458.9409, found: 1458.9412, correct isotope distribution.

Hexakis((triisopropylsilyl)ethynyl)-tetrabenzononacen (9a)

1a (50.0 mg, 28.2 µmol, 1.00 eq.) and **9** (25.4 mg, 84.5 µmol, 3.00 eq.) were added to a Schlenk flask under argon atmosphere. $Pd(P(tBu)_3)_2$ (1.4 mg, 2.81 µmol, 0.1 eq.) was added and the solids were dissolved in dry THF (10.0 mL) in a glovebox. The reaction mixture was stirred at 70 °C for 48 h, cooled to rt and the product was precipitated by dropwise adding the mixture to ACN. The suspension was filtered, and the dark green precipitate was washed with acetonitrile to yield the crude compound **9a** as a brown solid (15.0 mg, 8.52 µmol, 30%). Single crystal specimen were obtained by evaporation of a concentrated dichloromethane solution of the crude product. The compound was not soluble enough for ^{13}C NMR analysis.[9] ^{1}H NMR spectra were always contaminated. Mp: > 350 °C. IR (ATR): $\tilde{\nu}$ [cm^{-1}]: 2946, 2863, 2345, 2110, 1375, 1258, 1061, 1013, 881, 802, 729, 669, 639. HRMS (MALDI+, DCTB): m/z calcd. for $C_{120}H_{150}Si_6$: [M+] 1759.0348, found: 1759.0365, correct isotope distribution.

2 Characterization

2.1 NMR Spectroscopy

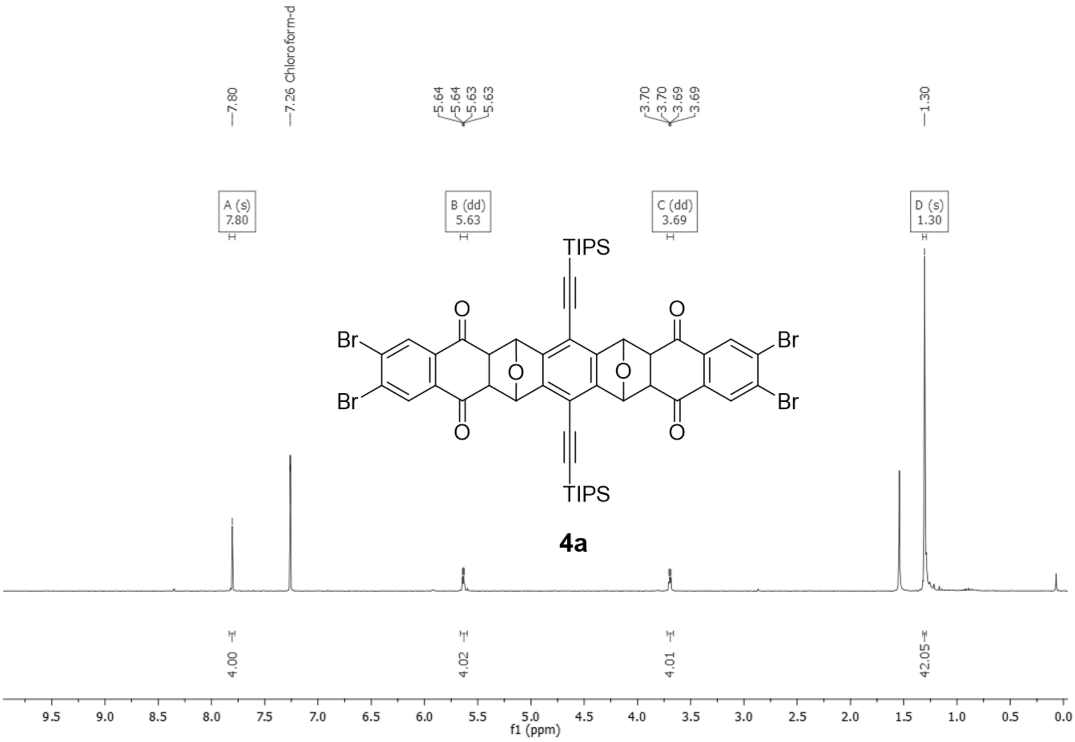

Figure S1. ^{1}H NMR spectrum (300 MHz, $CDCl_3$, 298 K) of **4a**.

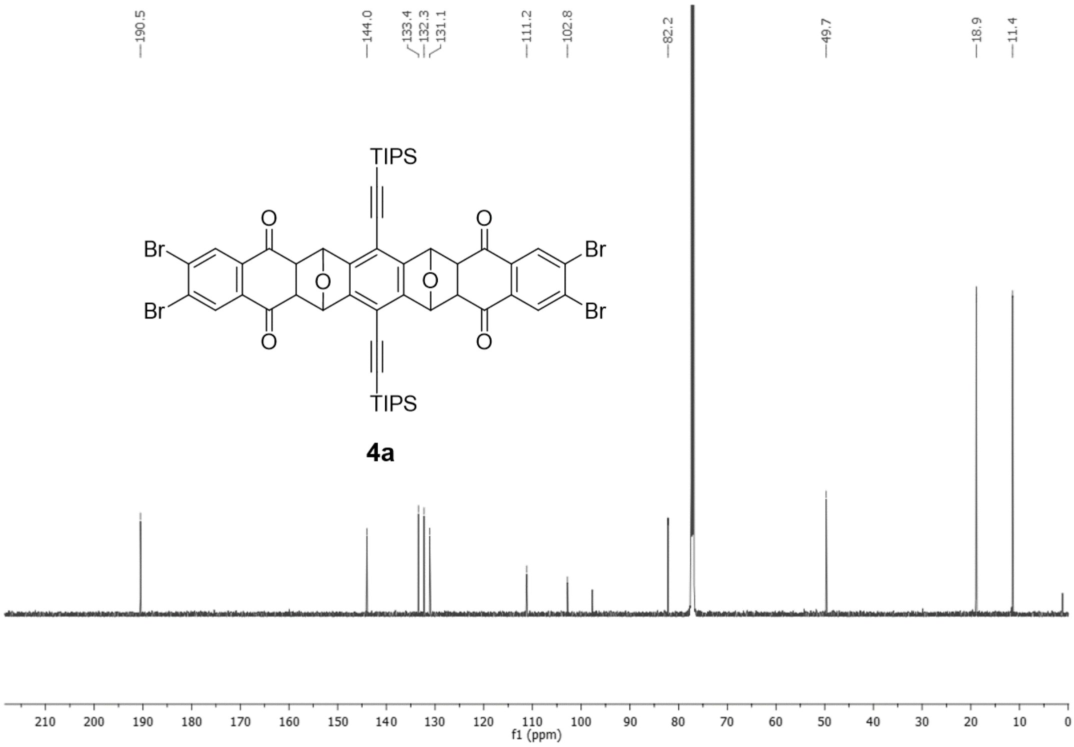

Figure S2. $^{13}C\{^{1}H\}$ NMR spectrum (151 MHz, $CDCl_3$, 298 K) of **4a**.

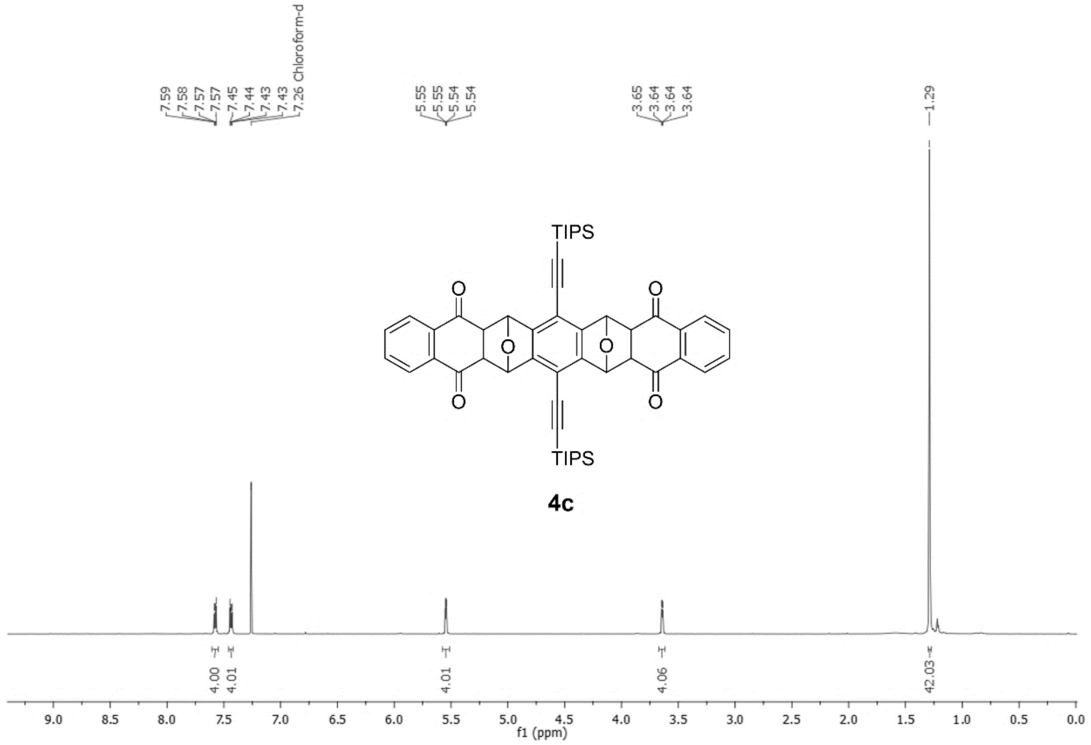

Figure S3. ^{1}H NMR spectrum (300 MHz, $CDCl_3$, 298 K) of **4c**.

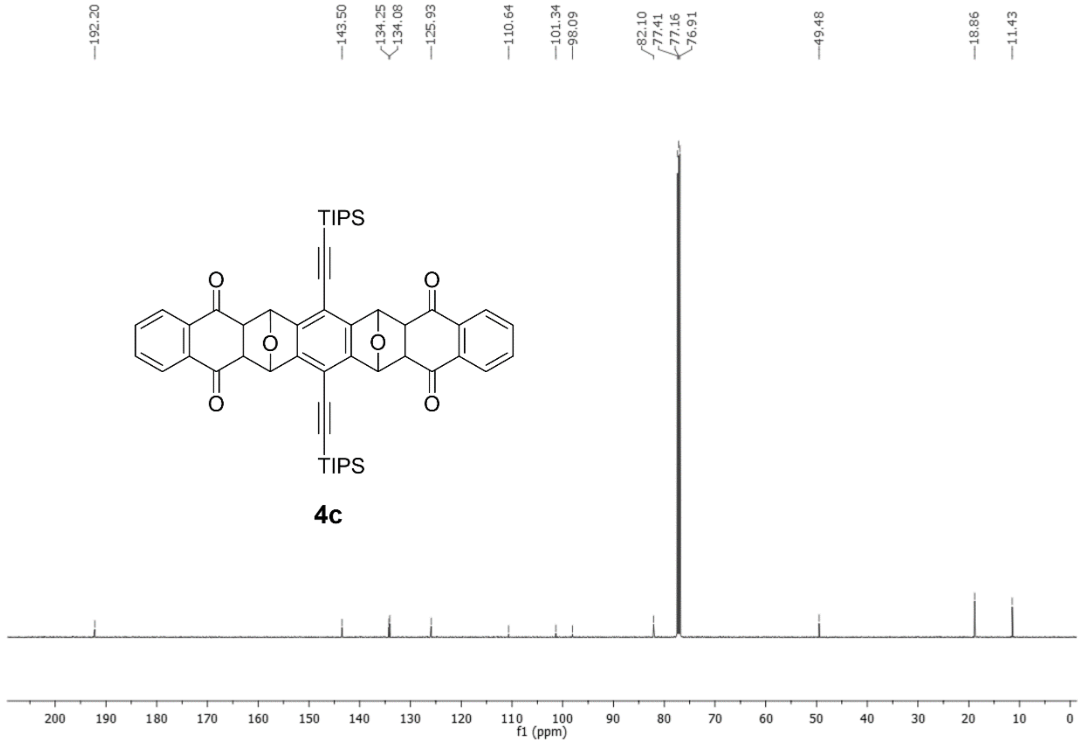

Figure S4. $^{13}C\{^{1}H\}$ NMR spectrum (126 MHz, $CDCl_3$, 298 K) of **4c.**

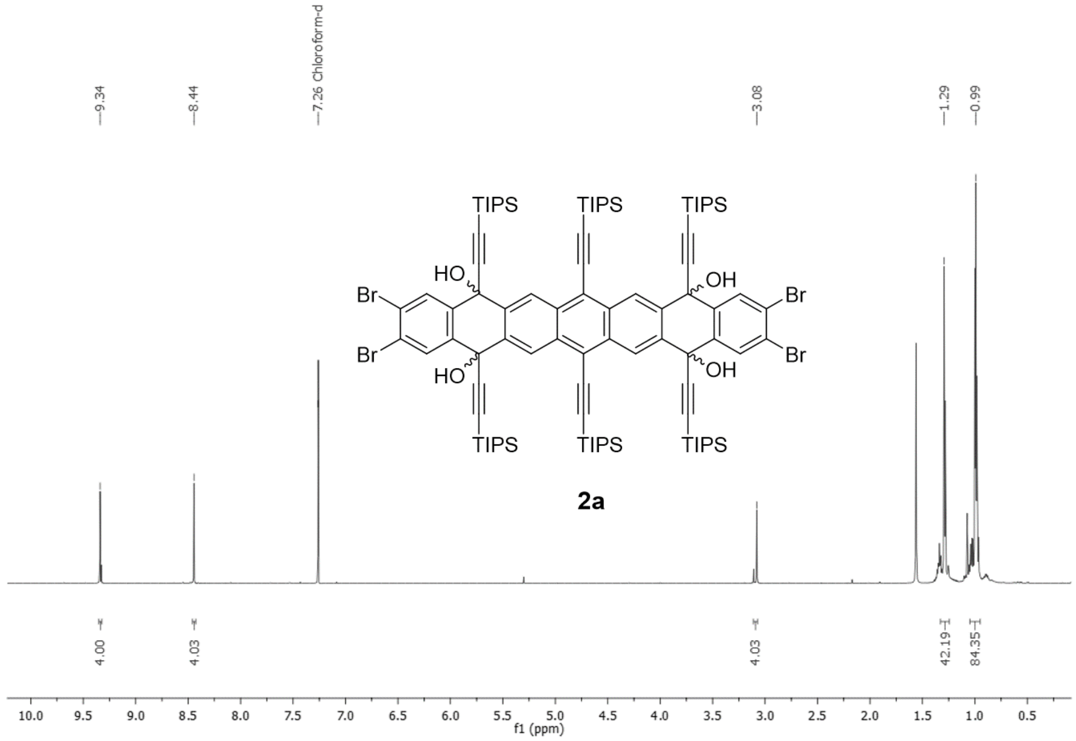

Figure S5. ^{1}H NMR spectrum (600 MHz, $CDCl_3$,298 K) of **2a**.

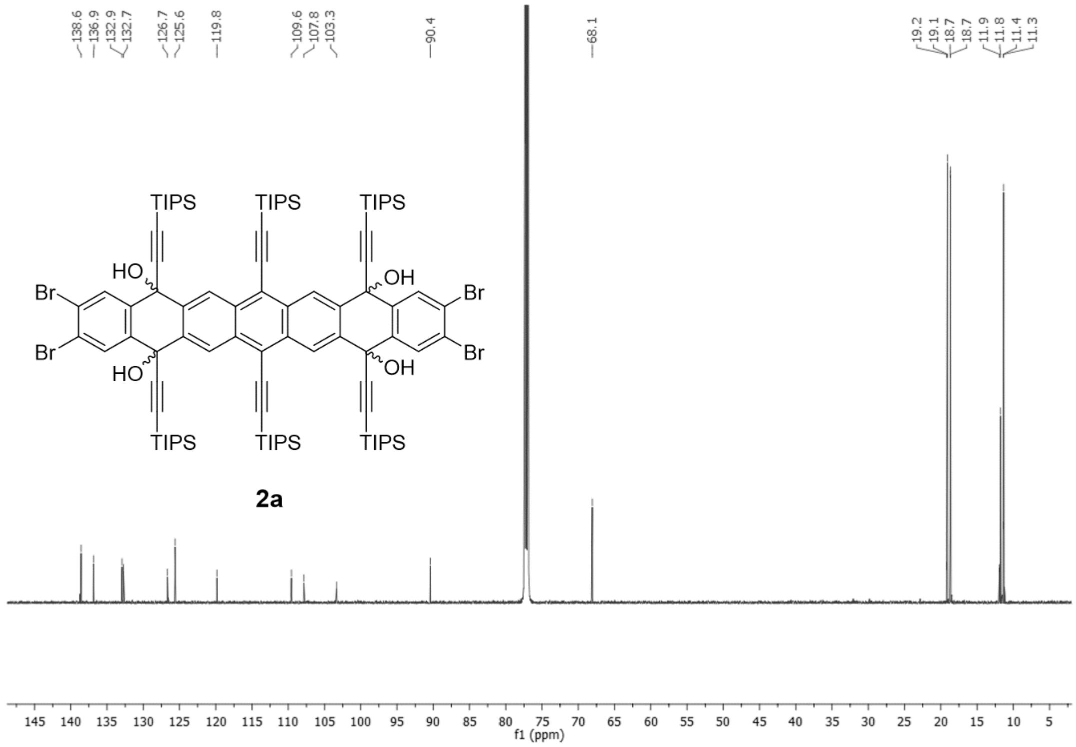

Figure S6. ^{13}C{^{1}H} NMR spectrum (151 MHz, $CDCl_3$, 298 K) of **2a**.

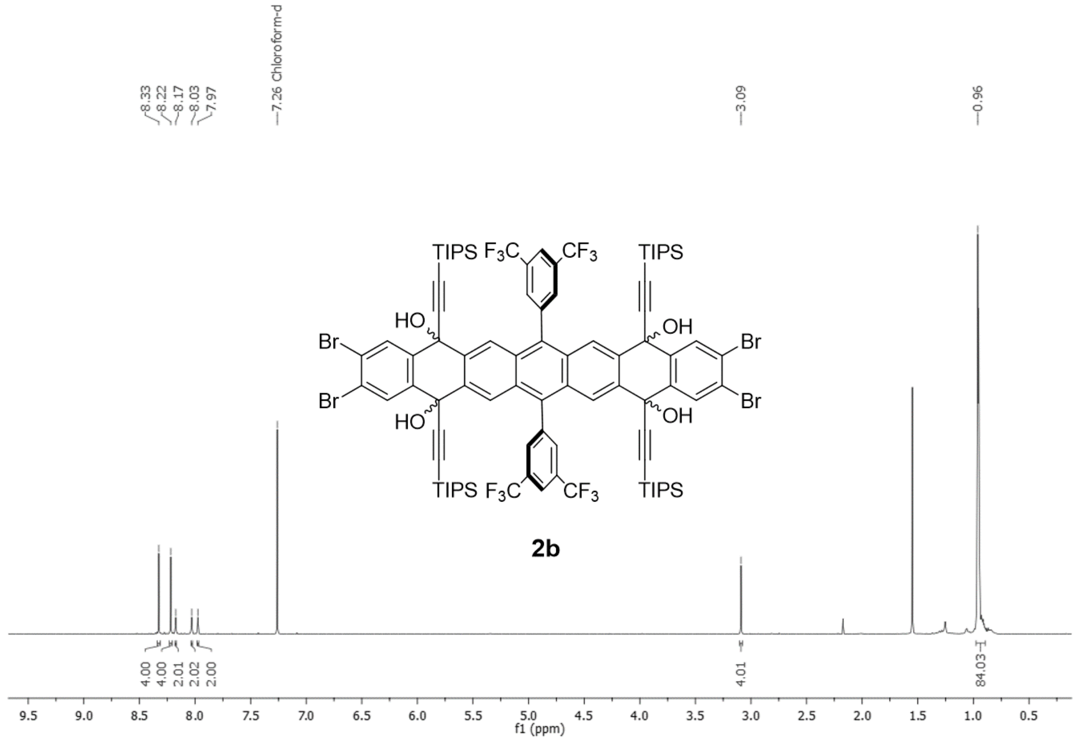

Figure S8. ^{1}H NMR spectrum (600 MHz, $CDCl_3$, 298 K) of **2b**.

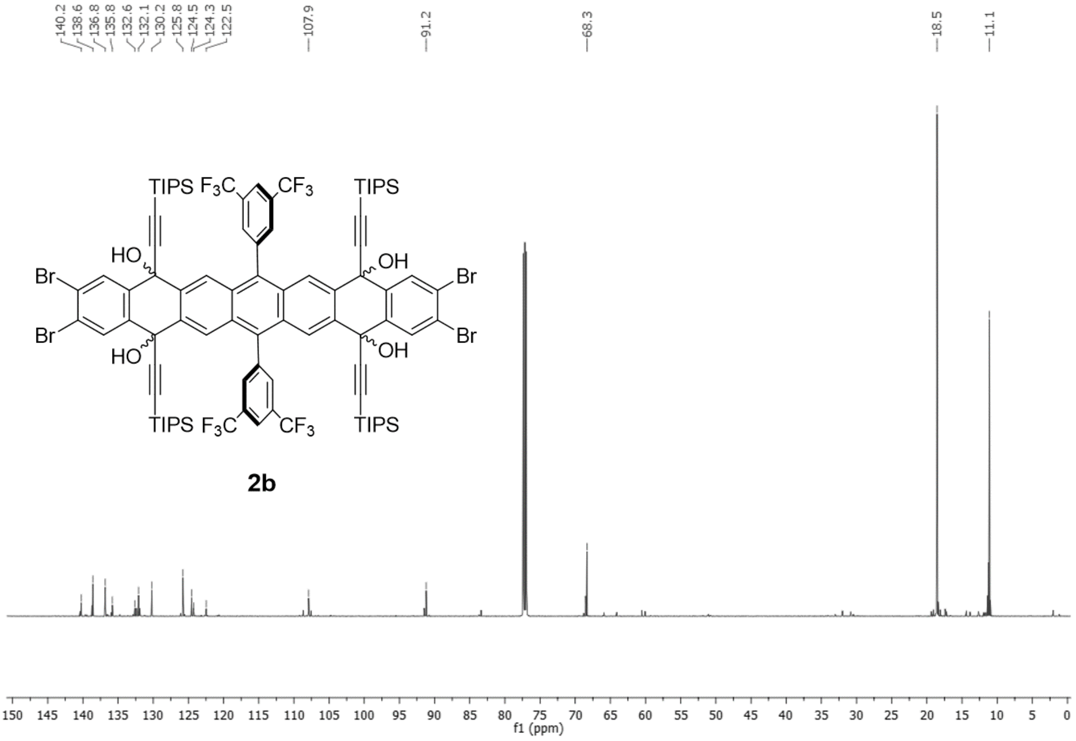

Figure S7. $^{13}C\{^{1}H\}$ NMR spectrum (151 MHz, $CDCl_3$, 298 K) of **2b**.

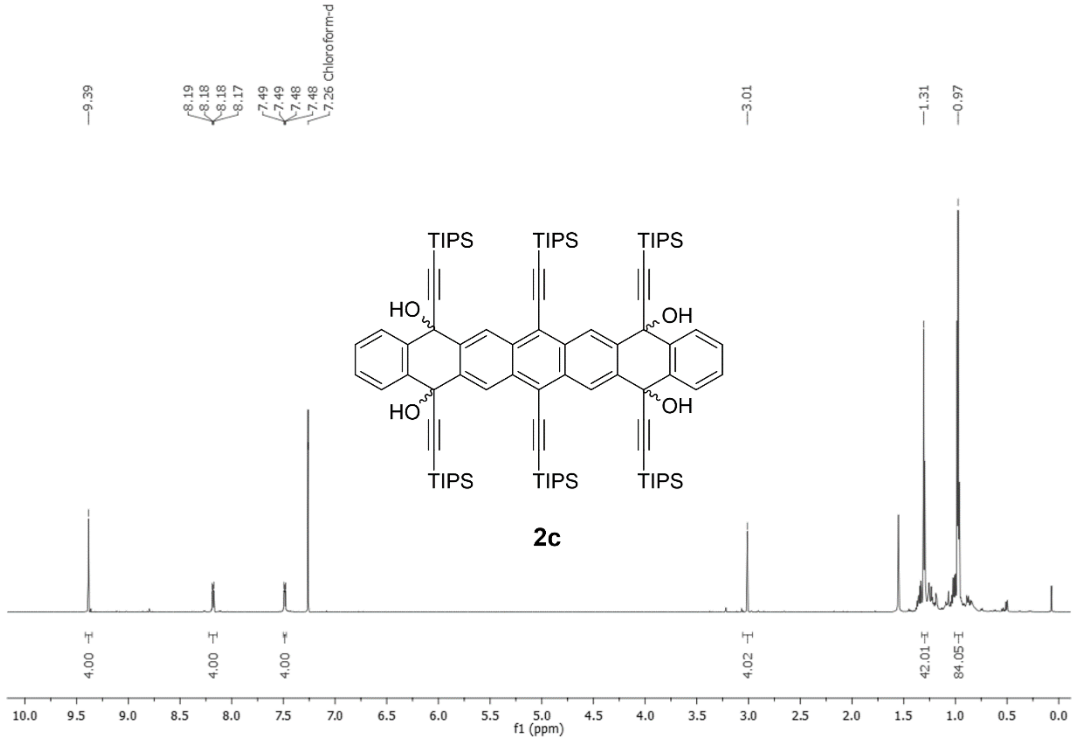

Figure S9. ^{1}H NMR spectrum (600 MHz, $CDCl_3$, 298 K) of **2c**.

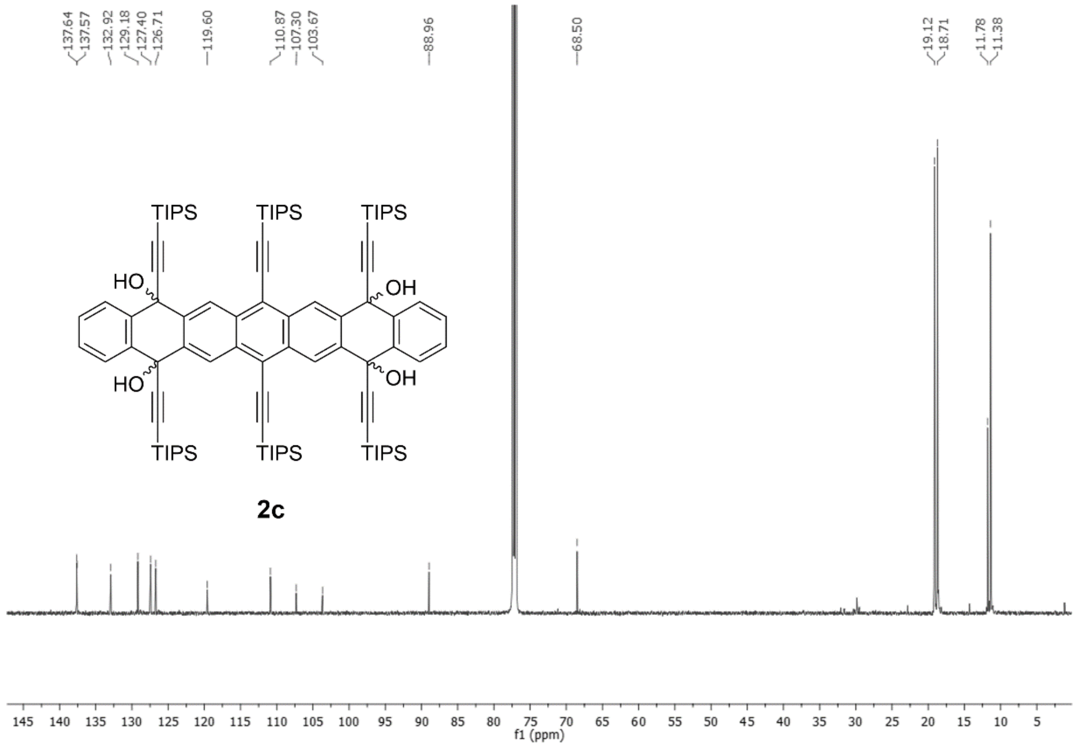

Figure S10. $^{13}C\{^{1}H\}$ NMR spectrum (151 MHz, $CDCl_3$, 298 K) of **2c.**

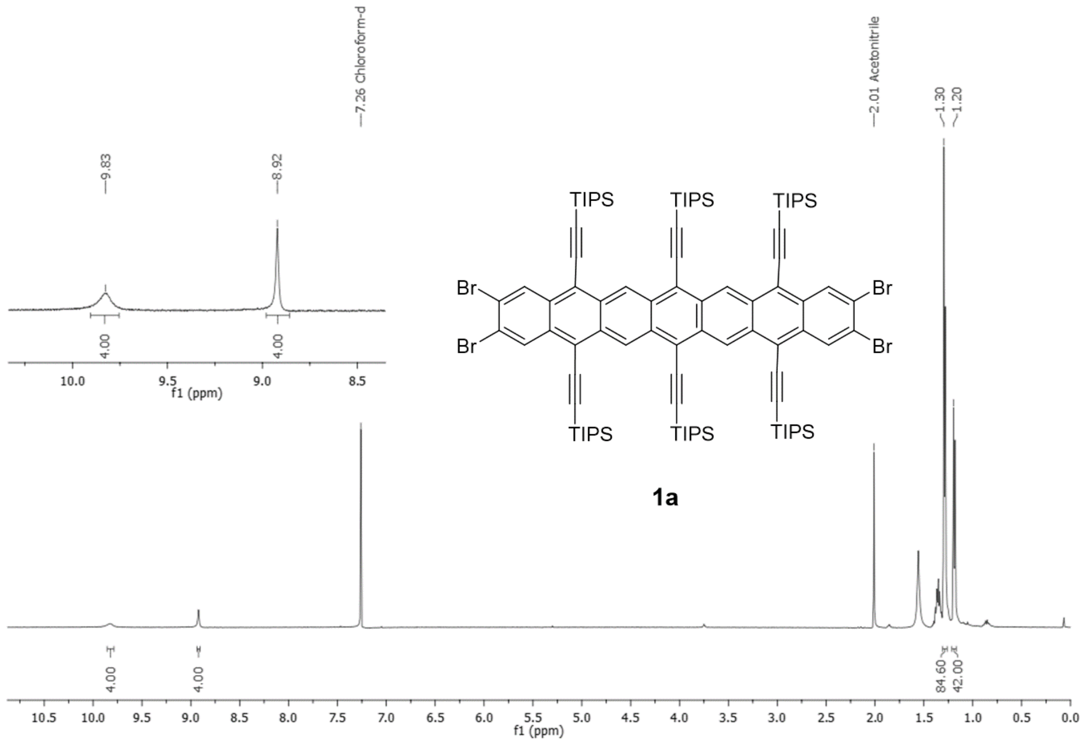

Figure S11. ^{1}H NMR spectrum (500 MHz, $CDCl_3$,298 K) of **1a**.

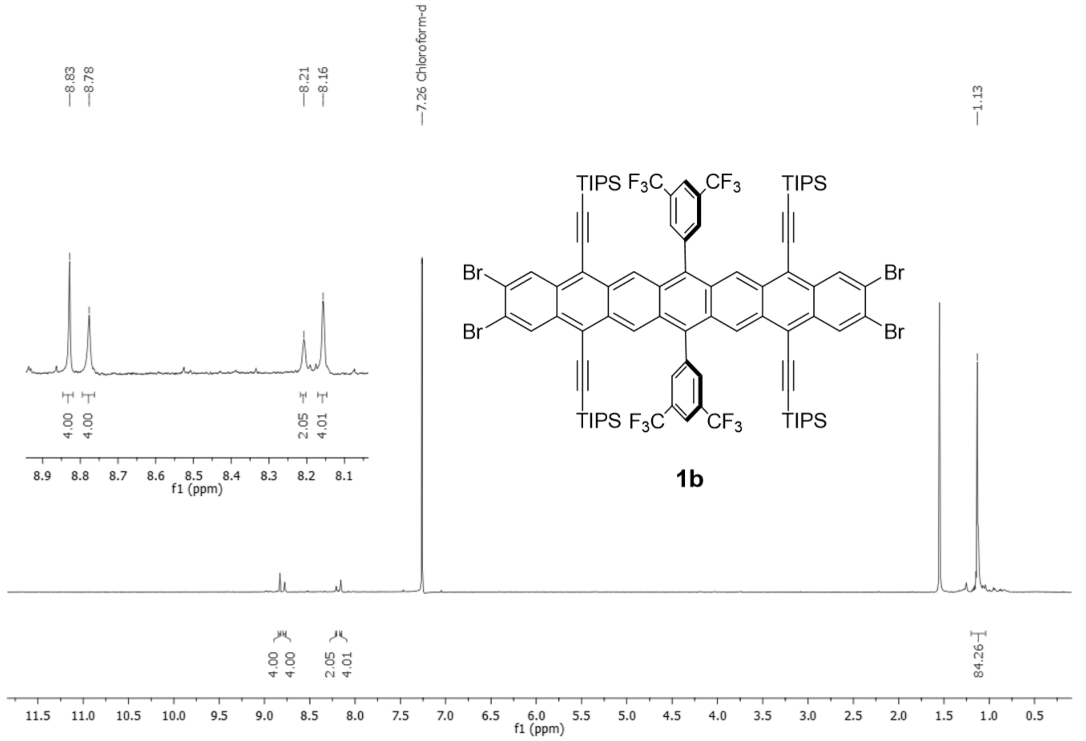

Figure S12. ^{1}H NMR spectrum (500 MHz, $CDCl_3$, 298 K) of **1b**.

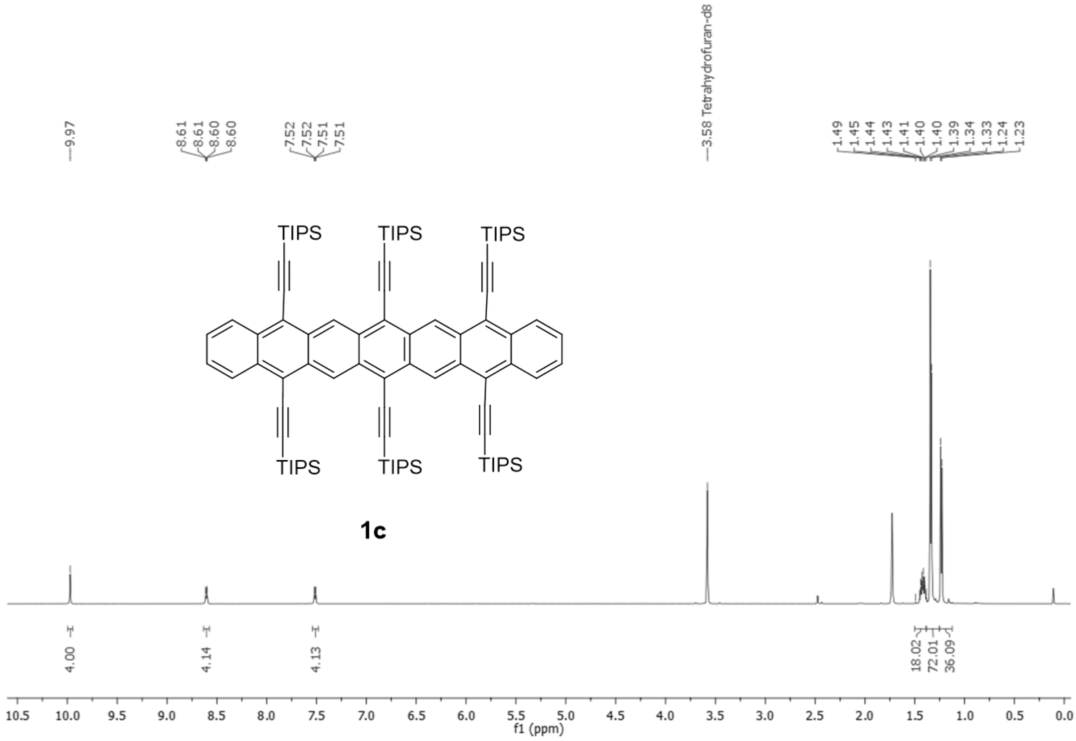

Figure S13. ^{1}H NMR spectrum (600 MHz, THF-d8, 298 K) of **1c.**

2.2 IR Spectroscopy

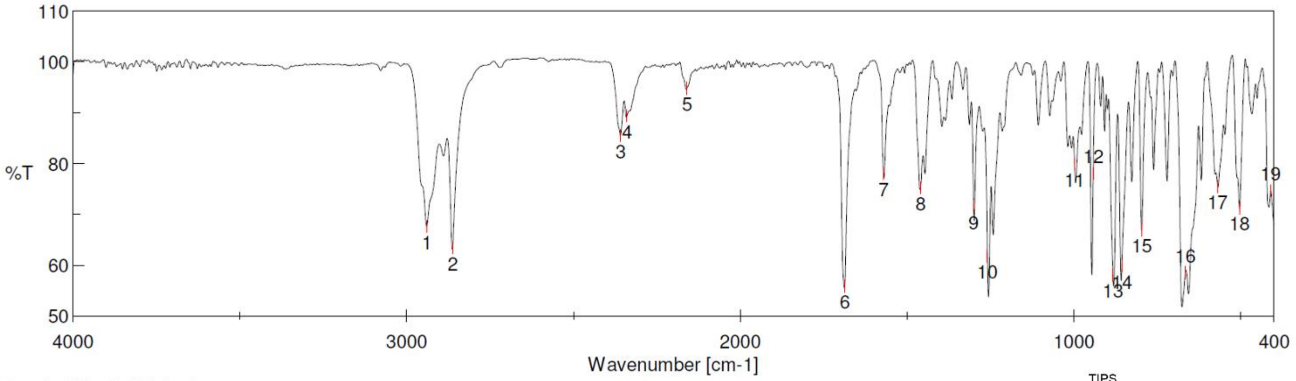

[Result of Peak Picking]

No.	Position	Intensity	No.	Position	Intensity	No.	Position	Intensity
1	2938.98	67.6073	2	2861.84	63.4236	3	2358.52	85.4417
4	2339.23	89.3302	5	2159.88	94.7086	6	1686.44	55.8821
7	1570.74	78.0291	8	1459.85	75.1841	9	1300.75	71.4649
10	1259.29	61.7579	11	997.982	79.8314	12	942.056	78.0072
13	883.238	57.9917	14	856.239	59.7864	15	796.457	66.8904
16	666.285	58.4853	17	568.898	75.4673	18	504.294	71.242
19	410.763	74.5618						

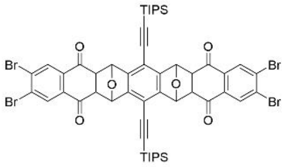

4a

Figure S14. IR spectrum of **4a.**

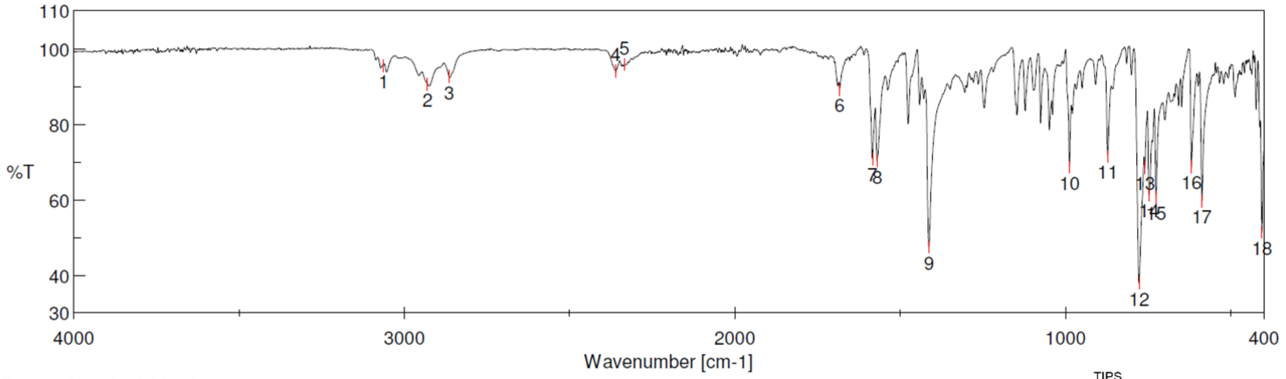

[Result of Peak Picking]

No.	Position	Intensity	No.	Position	Intensity	No.	Position	Intensity
1	3062.89	95.4676	2	2929.34	90.5553	3	2864.74	92.6092
4	2361.41	94.0859	5	2333.93	95.7661	6	1683.07	89.2081
7	1584.24	70.85	8	1569.77	70.222	9	1413.08	47.3988
10	987.857	68.6026	11	872.149	71.5723	12	777.172	37.924
13	759.816	68.4684	14	747.281	61.2016	15	726.068	60.5509
16	619.038	68.6464	17	587.218	59.6824	18	405.942	51.3527

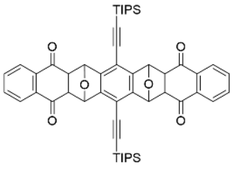

4c

Figure S15. IR spectrum of **4c.**

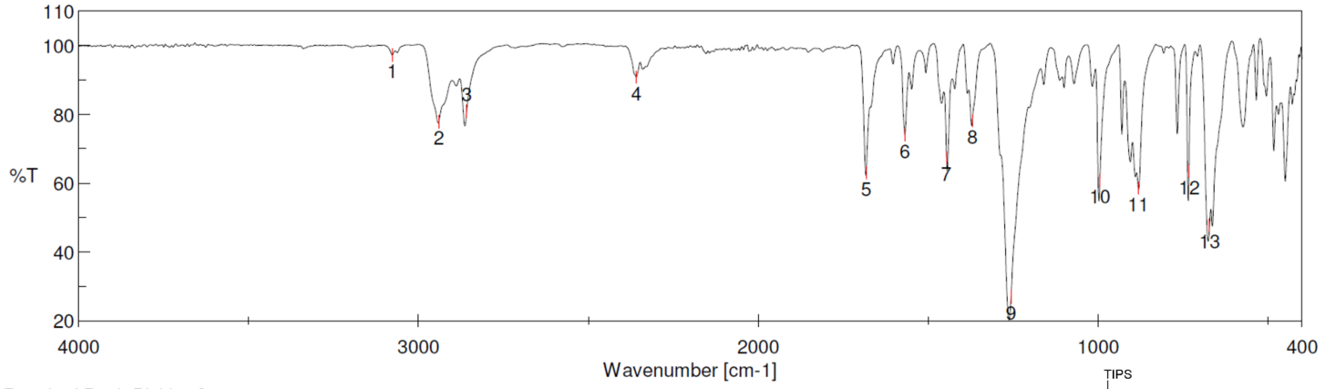

[Result of Peak Picking]

No.	Position	Intensity	No.	Position	Intensity	No.	Position	Intensity
1	3077.83	97.1636	2	2939.95	77.7876	3	2857.99	80.7892
4	2359.48	90.676	5	1682.59	62.9185	6	1568.81	73.9823
7	1446.35	67.355	8	1370.18	78.1752	9	1256.4	26.8443
10	995.089	60.7643	11	882.274	58.5903	12	732.817	63.4646
13	672.071	47.7486						

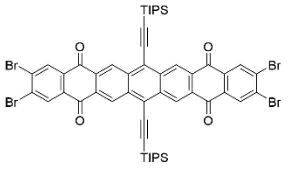

3a

Figure S16. IR spectrum of **3a**.

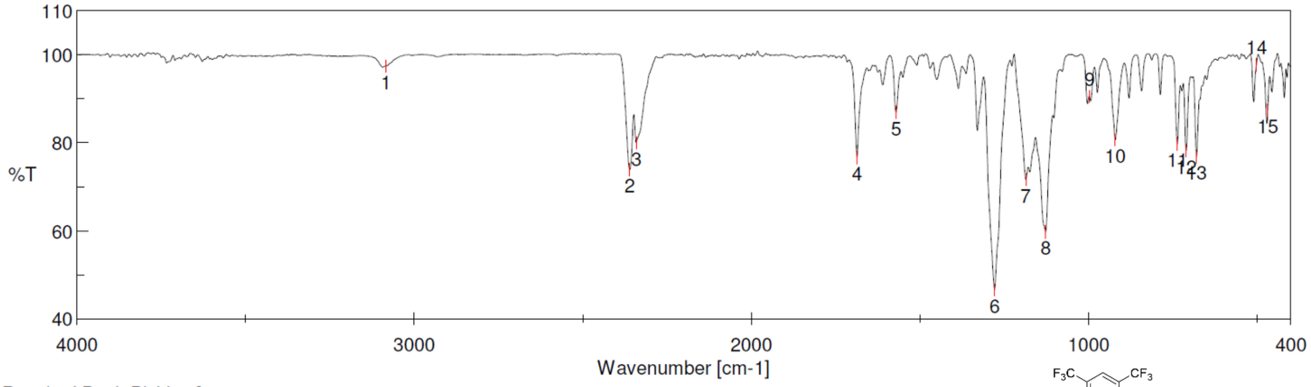

[Result of Peak Picking]

No.	Position	Intensity	No.	Position	Intensity	No.	Position	Intensity
1	3083.62	97.3815	2	2360.44	73.9006	3	2341.16	79.9535
4	1686.44	76.564	5	1570.74	86.9142	6	1278.57	46.5971
7	1186.01	71.5328	8	1127.19	59.6768	9	997.017	90.5246
10	920.843	80.6038	11	736.674	79.6508	12	710.64	78.109
13	679.785	76.7307	14	502.366	97.6797	15	468.617	87.3845

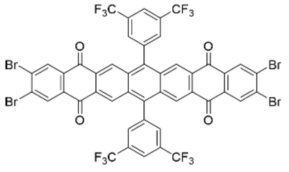

3b

Figure S17. IR spectrum of **3b**.

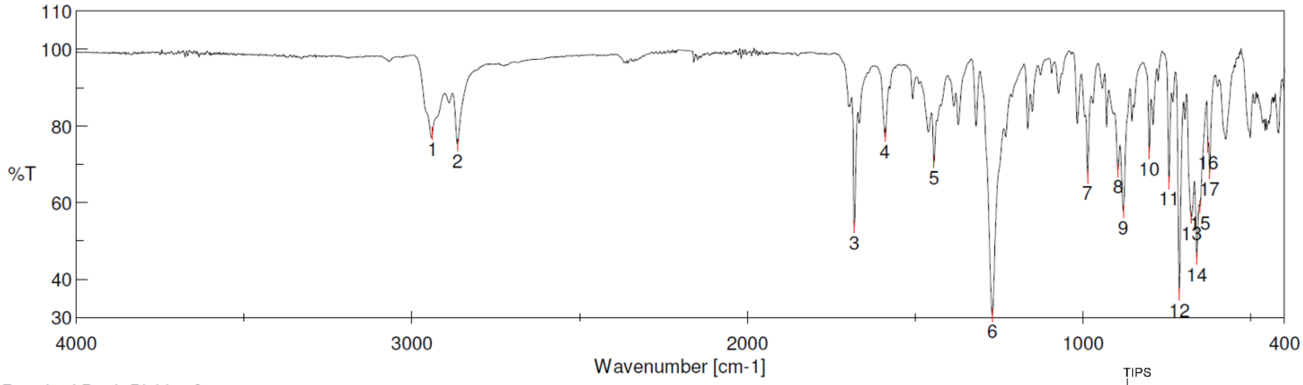

[Result of Peak Picking]

No.	Position	Intensity	No.	Position	Intensity	No.	Position	Intensity
1	2938.5	78.0132	2	2863.77	75.0508	3	1682.11	53.6376
4	1590.99	77.5413	5	1443.94	70.6043	6	1270.38	30.5085
7	986.411	66.5798	8	896.255	68.3151	9	880.345	57.5486
10	803.206	73.0173	11	743.906	65.2016	12	713.533	36.0655
13	677.374	56.1192	14	661.464	45.3564	15	651.822	58.8096
16	627.716	74.5958	17	622.895	67.7804			

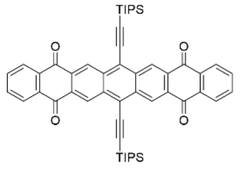

3c

Figure S18. IR spectrum of **3c.**

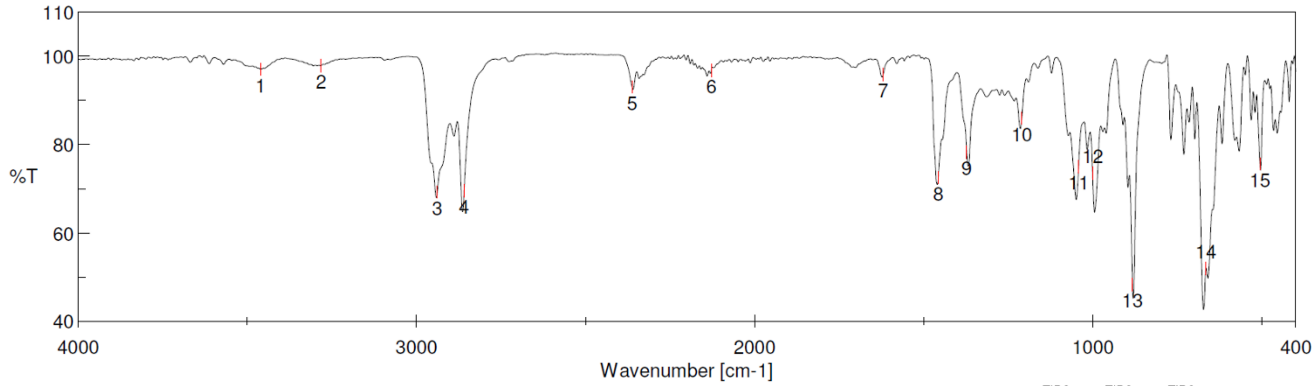

[Result of Peak Picking]

No.	Position	Intensity	No.	Position	Intensity	No.	Position	Intensity
1	3460.63	97.0616	2	3281.29	97.8471	3	2938.02	69.2326
4	2858.95	69.462	5	2362.37	93.0564	6	2128.06	96.7406
7	1619.91	95.8613	8	1456.96	72.4841	9	1373.07	78.2346
10	1210.11	85.89	11	1042.34	74.9018	12	1000.87	73.4719
13	883.238	48.2426	14	666.285	51.8553	15	506.223	75.6186

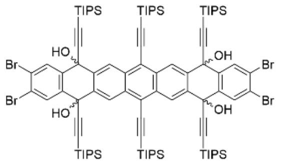

2a

Figure S19. IR spectrum of **2a.**

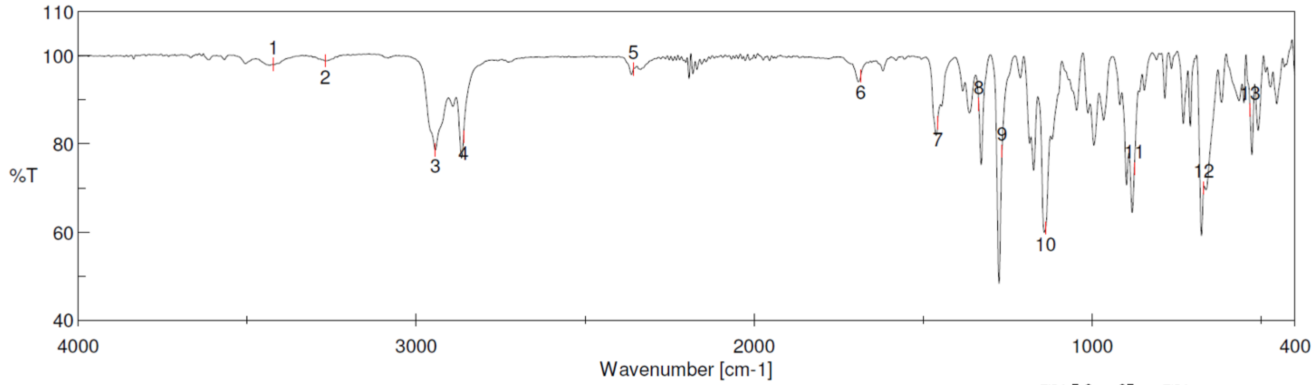

[Result of Peak Picking]

No.	Position	Intensity	No.	Position	Intensity	No.	Position	Intensity
1	3422.06	97.9735	2	3269.72	98.7952	3	2942.84	78.5458
4	2859.92	81.6218	5	2357.55	96.9056	6	1684.52	95.3825
7	1456.96	84.7059	8	1335.46	88.8915	9	1267	78.2928
10	1137.8	60.829	11	875.524	74.3698	12	670.142	69.9806
13	533.221	87.6913						

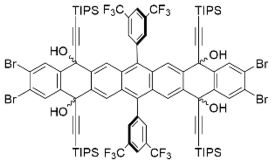

2b

Figure S20. IR spectrum of **2b.**

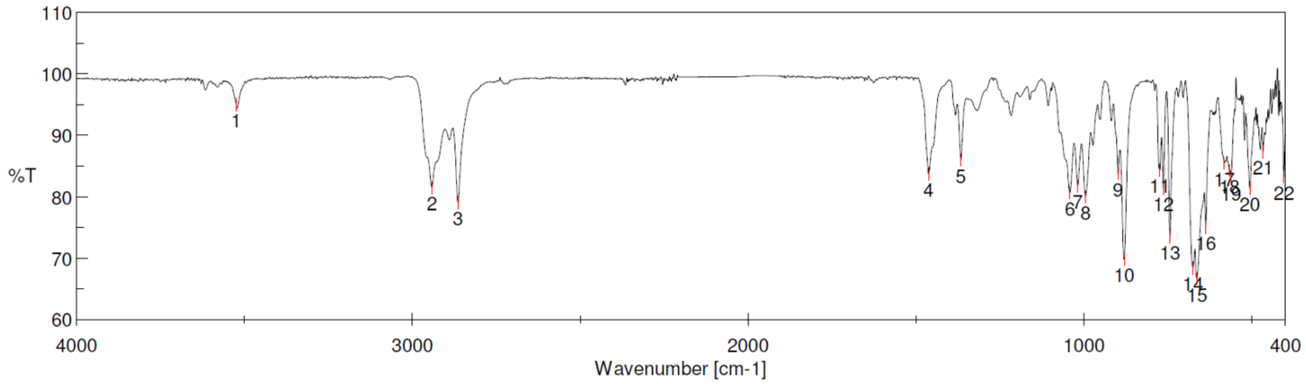

[Result of Peak Picking]

No.	Position	Intensity	No.	Position	Intensity	No.	Position	Intensity
1	3524.76	94.9501	2	2941.88	81.4746	3	2864.26	79.0145
4	1462.74	83.6612	5	1366.8	85.93	6	1042.34	80.6206
7	1018.71	81.7215	8	995.571	79.9965	9	898.666	83.7223
10	880.827	69.7642	11	774.279	84.3276	12	763.673	81.3417
13	743.906	73.4308	14	675.446	68.379	15	663.875	66.5815
16	637.358	75.0013	17	582.397	85.4163	18	566.969	84.2467
19	562.148	83.0642	20	506.223	81.3247	21	466.689	87.2916
22	404.978	83.2314						

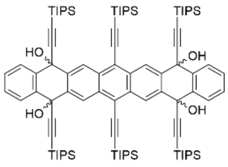

2c

Figure S21. IR spectrum of **2c.**

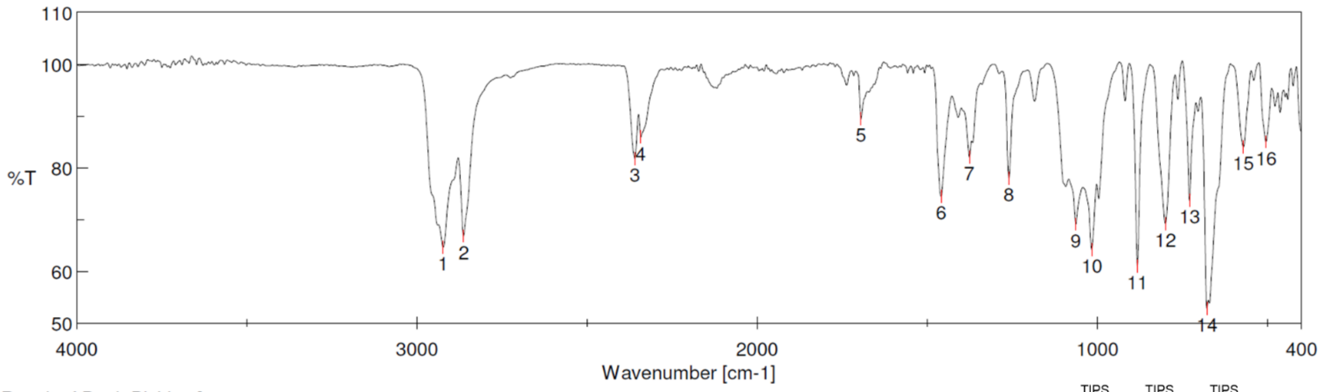

[Result of Peak Picking]

No.	Position	Intensity	No.	Position	Intensity	No.	Position	Intensity
1	2922.59	64.6377	2	2862.81	66.8197	3	2359.48	81.7514
4	2342.12	85.9244	5	1695.12	89.456	6	1458.89	74.4972
7	1375.96	82.0008	8	1260.25	77.9191	9	1063.55	69.1002
10	1016.3	64.1918	11	882.274	61.0777	12	800.314	69.205
13	728.961	73.6796	14	677.856	52.8274	15	570.826	84.0098
16	504.294	84.9942						

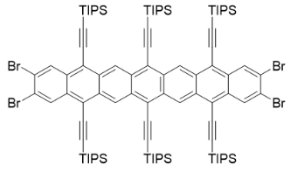

1a

Figure S22. IR spectrum of **1a**.

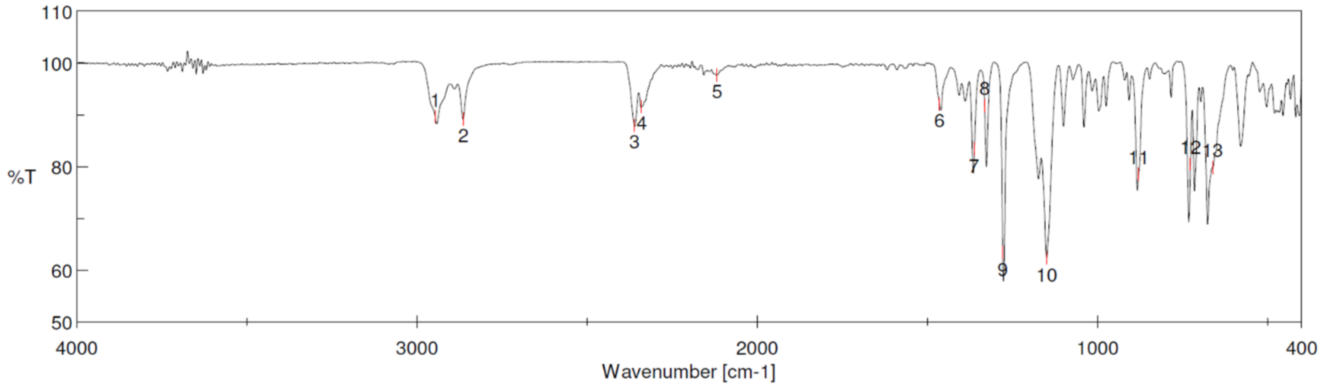

[Result of Peak Picking]

No.	Position	Intensity	No.	Position	Intensity	No.	Position	Intensity
1	2946.7	89.567	2	2863.77	89.2273	3	2361.41	87.9939
4	2339.23	91.5536	5	2118.42	97.6489	6	1464.67	92.1191
7	1361.5	83.3571	8	1331.61	91.7266	9	1278.57	63.3599
10	1149.37	62.3034	11	879.381	78.5004	12	727.032	80.4744
13	662.428	79.836						

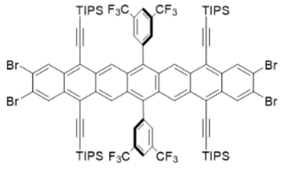

1b

Figure S23. IR spectrum of **1b**.

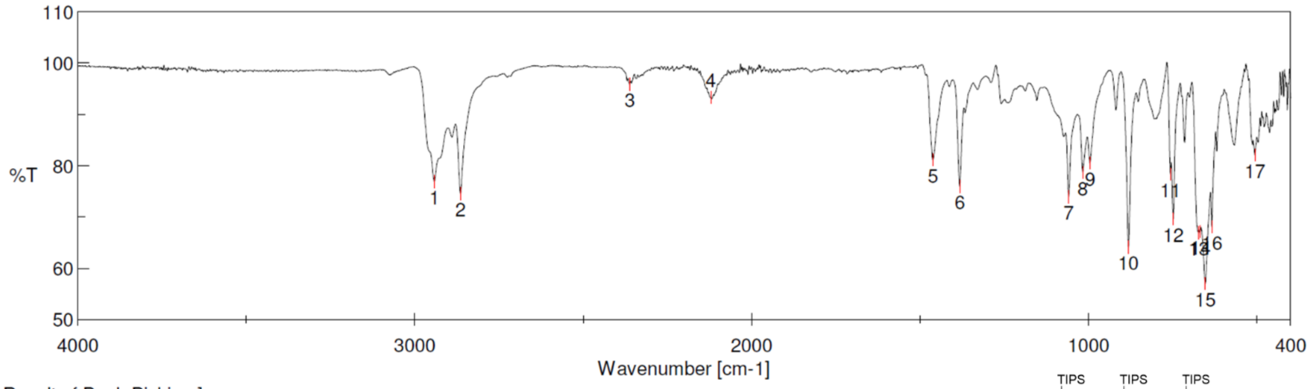

[Result of Peak Picking]

No.	Position	Intensity	No.	Position	Intensity	No.	Position	Intensity
1	2941.88	76.8807	2	2863.77	74.5201	3	2361.41	95.8501
4	2121.31	93.2603	5	1461.78	81.1331	6	1382.23	75.9401
7	1058.73	73.905	8	1016.78	78.6632	9	995.571	80.4564
10	881.792	64.0827	11	756.923	78.3228	12	748.727	69.6469
13	673.035	66.8579	14	668.214	67.1372	15	653.268	57.0242
16	633.984	68.0192	17	506.223	82.015			

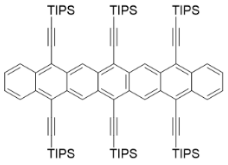

1c

Figure S24. IR spectrum of **1c**.

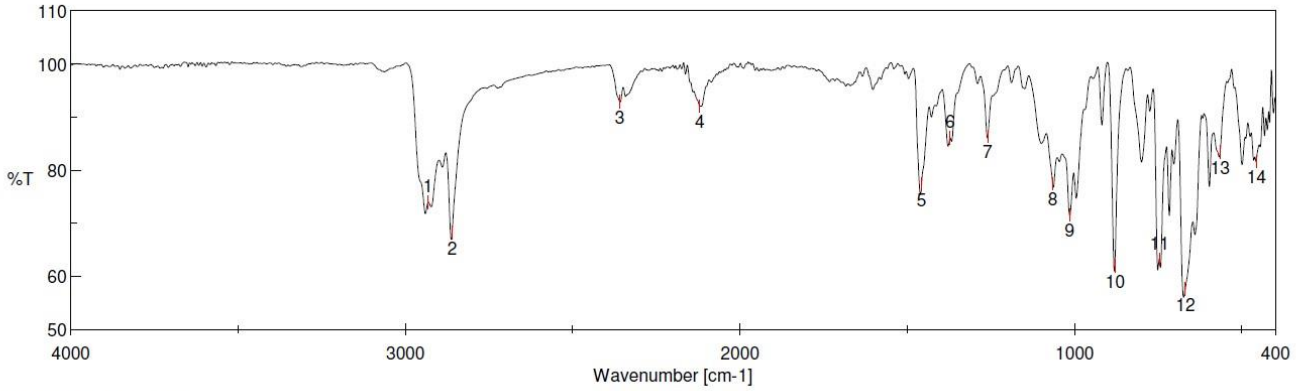

[Result of Peak Picking]

No.	Position	Intensity	No.	Position	Intensity	No.	Position	Intensity	No.	Position	Intensity
1	2932.23	73.8802	2	2859.92	68.236	3	2359.48	92.8871	4	2120.35	92.0265
5	1456.96	77.2737	6	1372.1	86.0107	7	1259.29	86.2998	8	1065.48	77.3973
9	1015.34	71.5189	10	879.381	61.9867	11	746.317	63.1797	12	669.178	57.5717
13	565.041	83.4018	14	457.047	81.6025						

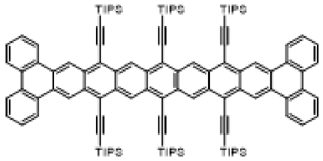

9a

Figure S25. IR spectrum of **9a**.

2.3 UV/vis Spectroscopy

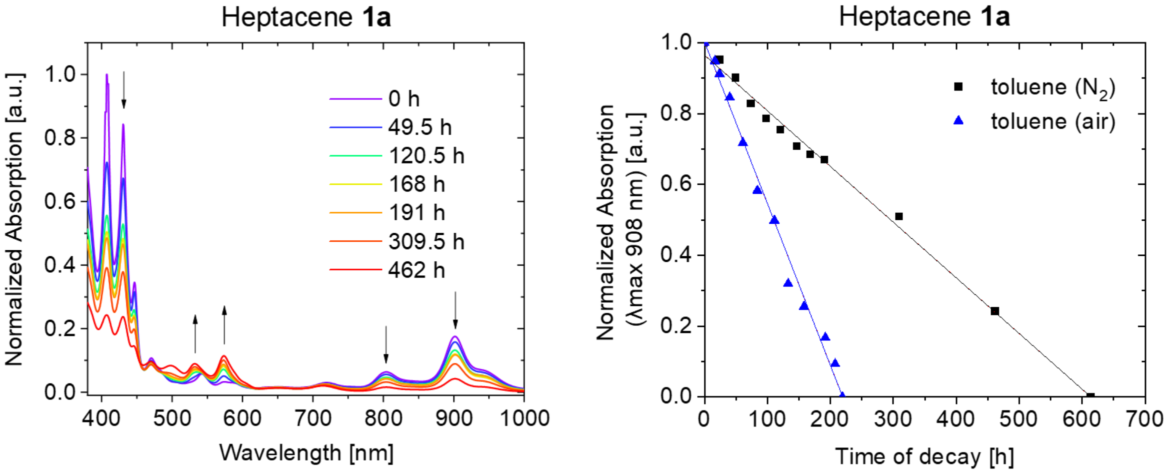

Figure S26. Change in UV/vis absorption intensity of **1a** under nitrogen atmosphere in toluene at room temperature and ambient light conditions (left), and time-dependent intensity decay (at λ_{max}) of **1a** (right).

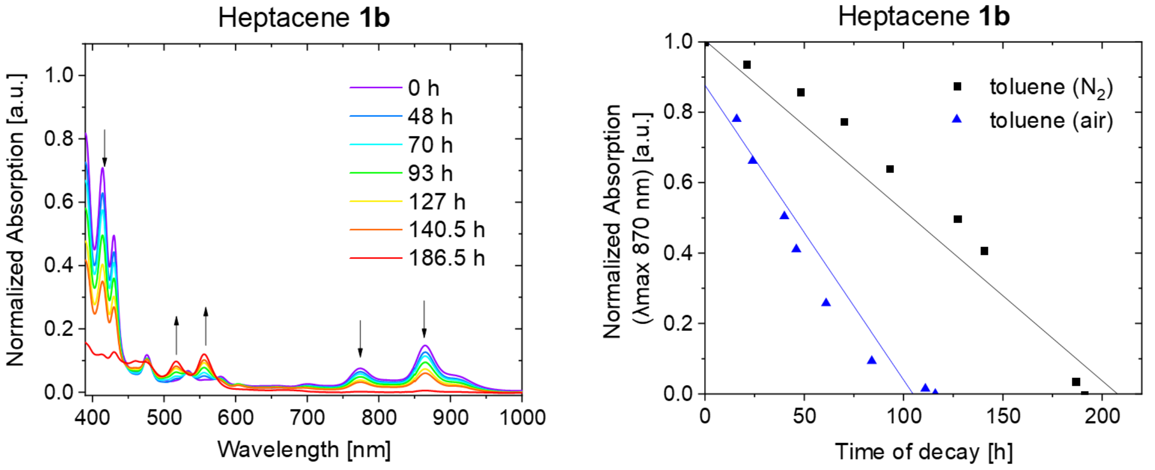

Figure S27. Change in UV/vis absorption intensity of **1b** under nitrogen atmosphere in toluene at room temperature and ambient light conditions (left), and time-dependent intensity decay (at λ_{max}) of **1b** (right).

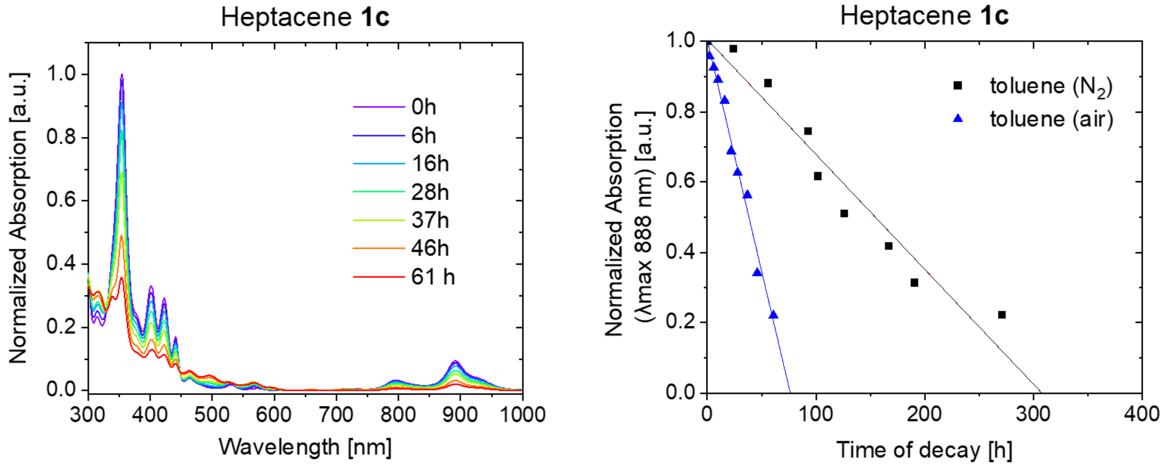

Figure S28. Change in UV/vis absorption intensity of **1c** under nitrogen atmosphere in toluene at room temperature and ambient light conditions (left), and time-dependent intensity decay (at λ_{max}) of **1c** (right).

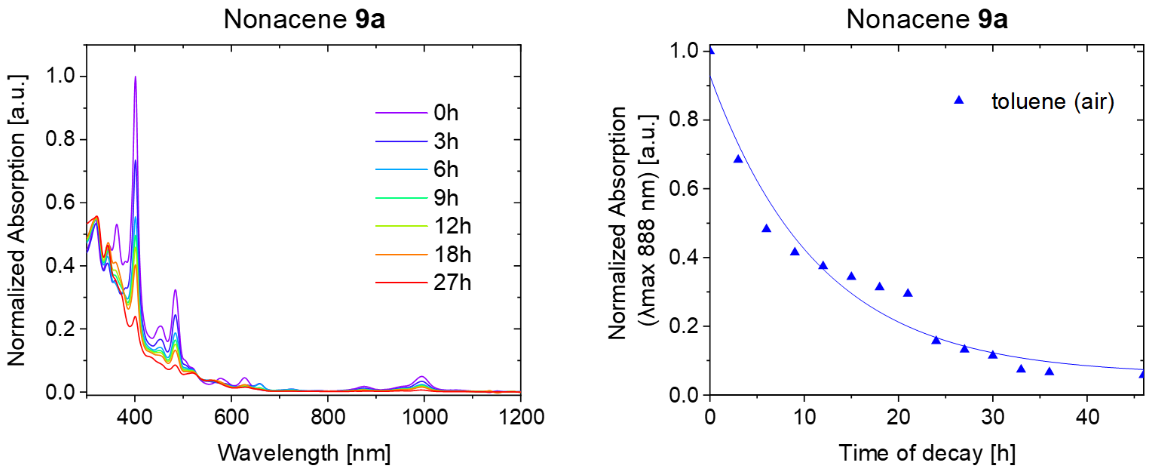

Figure S29. Change in UV/vis absorption intensity of **9a** under nitrogen atmosphere in toluene at room temperature and ambient light conditions (left), and time-dependent intensity decay (at λ_{max}) of **9a** (right).

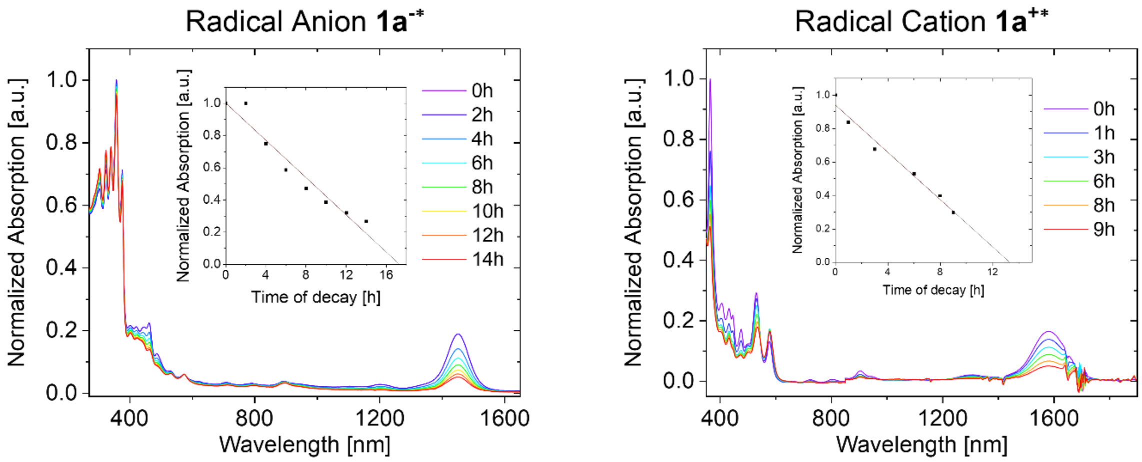

Figure S30. Change in UV/vis absorption intensity and the time dependent intensity decay (at λ_{max}) of **1a**$^{\bullet-}$ (left) and **1a**$^{\bullet+}$ (right) under ambient conditions in DCM at room temperature.

2.4 Cyclic Voltammetry

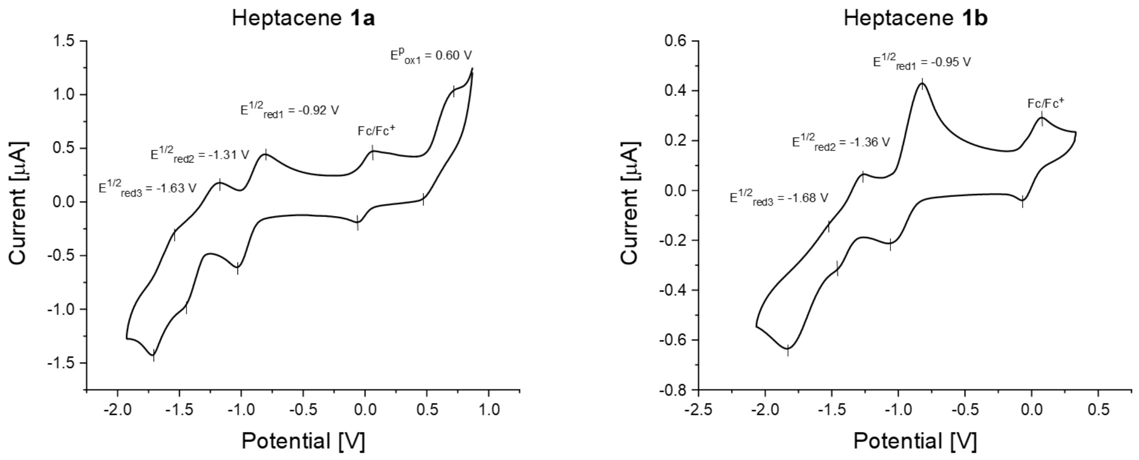

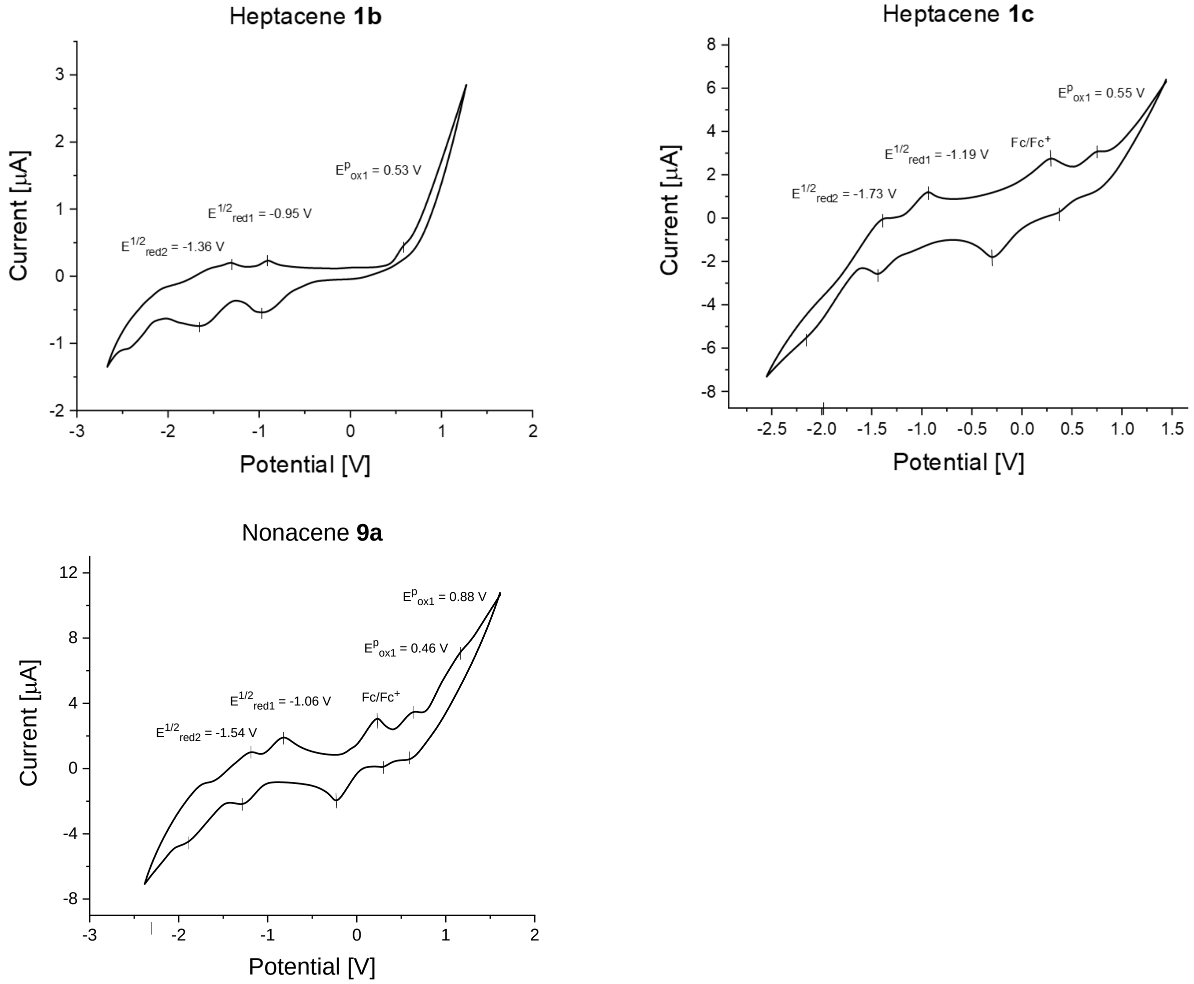

Figure S31. Cyclic voltammograms of **1a**, **1b**, **1c** and **9a** in CH_2Cl_2 using Bu_4NPF_6 as electrolyte, Pt as working electrode, Pt/Ti wire as counter electrode, silver wire as reference electrode and Fc/Fc^+ as internal standard at 0.2 Vs^{-1}.

2.5 Crystallographic Data

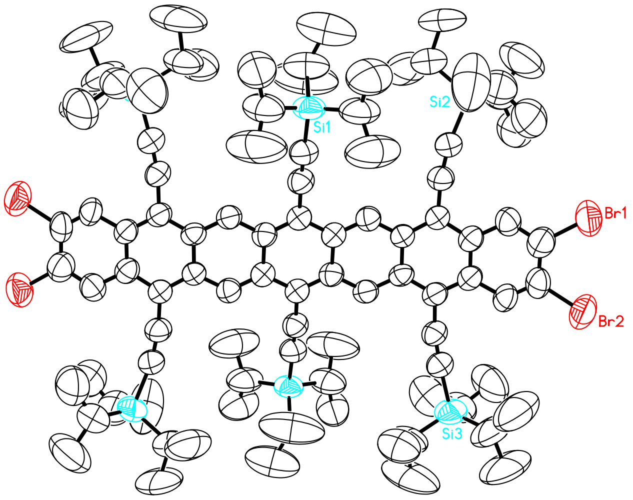

CCDC	2141664	
Empirical formula	$C_{96}H_{134}Br_4Si_6$	
Formula weight	1776.20	
Temperature	200(2) K	
Wavelength	1.54178 Å	
Crystal system	monoclinic	
Space group	$P2_1/c$	
Z	2	
Unit cell dimensions	a = 19.5958(11) Å	α = 90 deg.
	b = 16.0258(10) Å	β = 90.711(5) deg.
	c = 15.5121(10) Å	γ = 90 deg.
Volume	4871.0(5) $Å^3$	
Density (calculated)	1.21 g/cm^3	
Absorption coefficient	3.03 mm^{-1}	
Crystal shape	plank	
Crystal size	0.090 x 0.057 x 0.020 mm^3	
Crystal colour	brown	
Theta range for data collection	3.6 to 62.5 deg.	
Index ranges	$-22\leq h\leq 20$, $-17\leq k\leq 15$, $-17\leq l\leq 11$	
Reflections collected	24207	
Independent reflections	7240 (R(int) = 0.0508)	
Observed reflections	4186 ($I > 2\sigma(I)$)	
Absorption correction	Semi-empirical from equivalents	
Max. and min. transmission	4.65 and 0.76	
Refinement method	Full-matrix least-squares on F^2	
Data/restraints/parameters	7240 / 0 / 496	
Goodness-of-fit on F^2	1.03	
Final R indices (I>2sigma(I))	R1 = 0.059, wR2 = 0.121	
Largest diff. peak and hole	0.45 and -0.41 $eÅ^{-3}$	

Figure S32. Crystal structure, crystal data and structure refinement of **1a**.

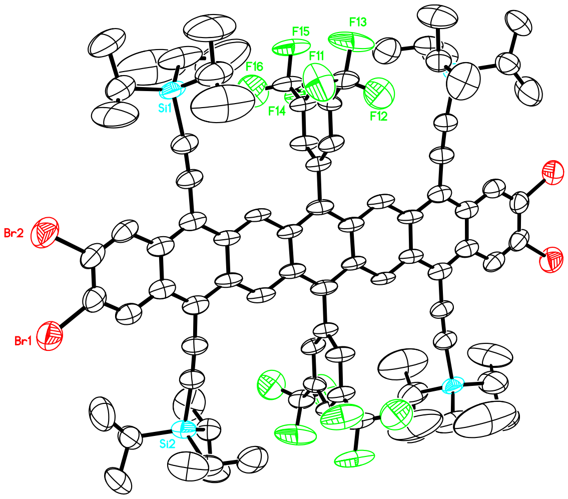

CCDC	2141665
Empirical formula	$C_{90}H_{98}Br_4F_{12}Si_4$
Formula weight	1839.68
Temperature	200(2) K
Wavelength	1.54178 Å
Crystal system	monoclinic
Space group	$P2_1/c$
Z	2
Unit cell dimensions	a = 19.2284(17) Å α = 90 deg.
	b = 15.1682(9) Å β =101.380(7) deg.
	c = 15.4726(13) Å γ = 90 deg.
Volume	4424.0(6) $Å^3$
Density (calculated)	1.38 g/cm^3
Absorption coefficient	3.31 mm^{-1}
Crystal shape	plate
Crystal size	0.058 x 0.056 x 0.015 mm^3
Crystal colour	green
Theta range for data collection	3.7 to 50.4 deg.
Index ranges	-19≤h≤18, -11≤k≤15, -15≤l≤15
Reflections collected	26331
Independent reflections	4625 (R(int) = 0.1113)
Observed reflections	2724 (I > 2σ(I))
Absorption correction	Semi-empirical from equivalents
Max. and min. transmission	1.47 and 0.57
Refinement method	Full-matrix least-squares on F^2
Data/restraints/parameters	4625 / 857 / 524
Goodness-of-fit on F^2	1.03
Final R indices (I>2sigma(I))	R1 = 0.069, wR2 = 0.140
Largest diff. peak and hole	0.52 and -0.29 $eÅ^{-3}$

Figure S33. Crystal structure, crystal data and structure refinement of **1b**.

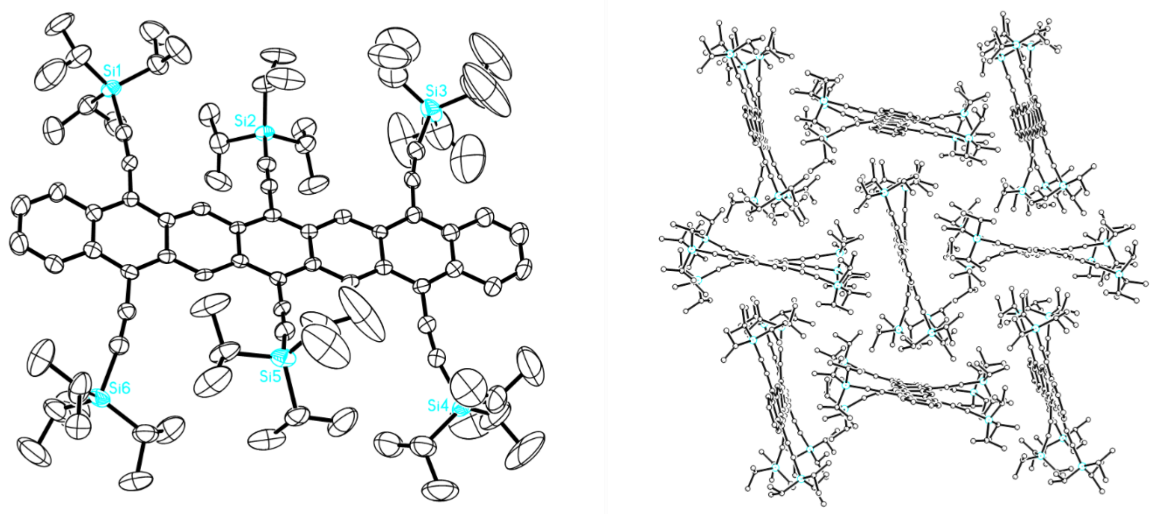

CCDC	2141666	
Empirical formula	$C_{96}H_{138}Si_6$	
Formula weight	1460.60	
Temperature	200(2) K	
Wavelength	1.54178 Å	
Crystal system	monoclinic	
Space group	Pn	
Z	4	
Unit cell dimensions	a = 15.2620(3) Å	α = 90 deg.
	b = 15.8514(4) Å	β = 98.506(1) deg.
	c = 38.2674(7) Å	γ = 90 deg.
Volume	9156.0(3) $Å^3$	
Density (calculated)	1.06 g/cm^3	
Absorption coefficient	1.16 mm^{-1}	
Crystal shape	plate	
Crystal size	0.211 x 0.185 x 0.030 mm^3	
Crystal colour	brown	
Theta range for data collection	3.6 to 66.9 deg.	
Index ranges	$-17 \le h \le 12$, $-15 \le k \le 18$, $-38 \le l \le 45$	
Reflections collected	47178	
Independent reflections	19256 (R(int) = 0.0485)	
Observed reflections	11282 ($I > 2\sigma(I)$)	
Absorption correction	Semi-empirical from equivalents	
Max. and min. transmission	1.00 and 0.68	
Refinement method	Full-matrix least-squares on F^2	
Data/restraints/parameters	19256 / 7682 / 1965	
Goodness-of-fit on F^2	0.95	
Final R indices (I>2sigma(I))	R1 = 0.056, wR2 = 0.131	
Absolute structure parameter	0.23(2)	
Largest diff. peak and hole	0.44 and -0.23 $eÅ^{-3}$	

Figure S34. Crystal structure, crystal data and structure refinement of **1c**.

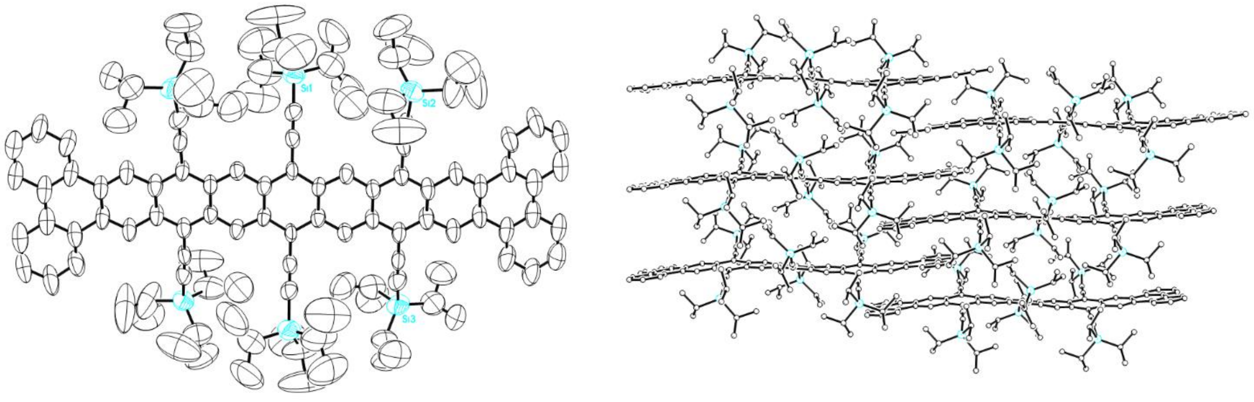

CCDC	2141667
Empirical formula	$C_{120}H_{150}Si_6$
Formula weight	1760.93
Temperature	200(2) K
Wavelength	1.54178 Å
Crystal system	triclinic
Space group	$P\bar{1}$
Z	1
Unit cell dimensions	a = 8.9378(8) Å α = 75.520(6) deg.
	b = 16.9975(12) Å β = 85.032(7) deg.
	c = 18.2179(15) Å γ = 86.472(7) deg.
Volume	2667.4(4) $Å^3$
Density (calculated)	1.10 g/cm^3
Absorption coefficient	1.08 mm^{-1}
Crystal shape	brick
Crystal size	0.068 x 0.038 x 0.034 mm^3
Crystal colour	brown
Theta range for data collection	3.2 to 41.6 deg.
Index ranges	$-7\leq h\leq 7$, $-14\leq k\leq 9$, $-15\leq l\leq 15$
Reflections collected	10178
Independent reflections	3515 (R(int) = 0.0897)
Observed reflections	1776 ($I > 2\sigma(I)$)
Absorption correction	Semi-empirical from equivalents
Max. and min. transmission	1.34 and 0.72
Refinement method	Full-matrix least-squares on F^2
Data/restraints/parameters	3515 / 1581 / 656
Goodness-of-fit on F^2	1.02
Final R indices (I>2sigma(I))	R1 = 0.097, wR2 = 0.234
Largest diff. peak and hole	0.28 and -0.20 $eÅ^{-3}$

Figure S35. Crystal structure, crystal data and structure refinement of **9a**.

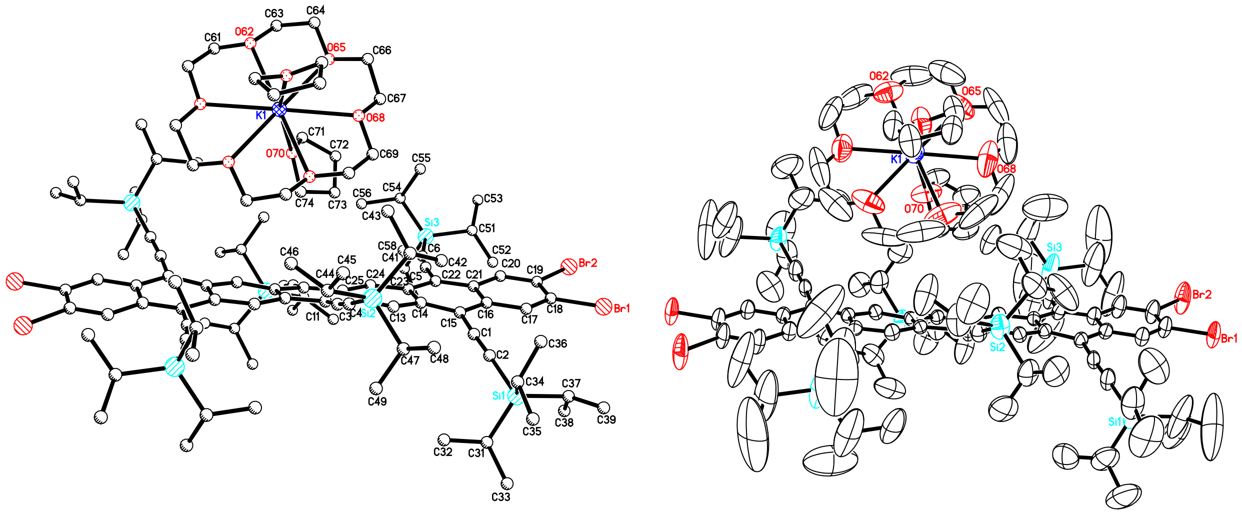

CCDC	2141668	
Empirical formula	$C_{116}H_{174}Br_4KO_8Si_6$	
Formula weight	2223.82	
Temperature	200(2) K	
Wavelength	0.71073 Å	
Crystal system	monoclinic	
Space group	C2/c	
Z	4	
Unit cell dimensions	a = 25.1931(10) Å	α = 90 deg.
	b = 27.8869(11) Å	β = 106.067(1) deg.
	c = 19.7506(8) Å	γ = 90 deg.
Volume	13333.9(9) $Å^3$	
Density (calculated)	1.11 g/cm^3	
Absorption coefficient	1.34 mm^{-1}	
Crystal shape	plank	
Crystal size	0.155 x 0.075 x 0.054 mm^3	
Crystal colour	brown	
Theta range for data collection	1.4 to 25.5 deg.	
Index ranges	$-30 \le h \le 30$, $-33 \le k \le 33$, $-23 \le l \le 23$	
Reflections collected	65585	
Independent reflections	12374 (R(int) = 0.0491)	
Observed reflections	7605 ($I > 2\sigma(I)$)	
Absorption correction	Semi-empirical from equivalents	
Max. and min. transmission	0.89 and 0.84	
Refinement method	Full-matrix least-squares on F^2	
Data/restraints/parameters	12374 / 0 / 609	
Goodness-of-fit on F^2	1.03	
Final R indices (I>2sigma(I))	R1 = 0.060, wR2 = 0.148	
Largest diff. peak and hole	0.83 and -1.31 $eÅ^{-3}$	

Figure S36. Crystal structure, crystal data and structure refinement of **1a**$^{\cdot-}$.

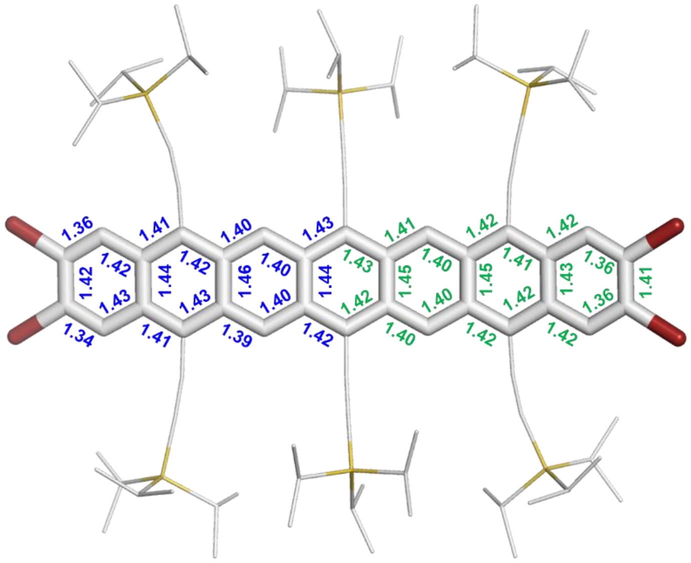

Figure S37: X-ray single crystal structure (top view) including bond lengths (in Å) of **1a** (blue) and the anionic form **1a**$^{\cdot-}$ (green). Determined by crystal analysis.

2.6 Analysis of Decomposition Products

A freshly prepared solution of **1a** in anhydrous THF under inert atmosphere was exposed to ambient conditions (light and air) for several days at rt. Afterwards the solvent was evaporated and the solid was analyzed via MALDI mass spectrometry. Formation of di-oxo-adducts is evident (position of oxidation was assigned via UV/vis spectroscopy as bands generated are characteristic of tetracene fragments).

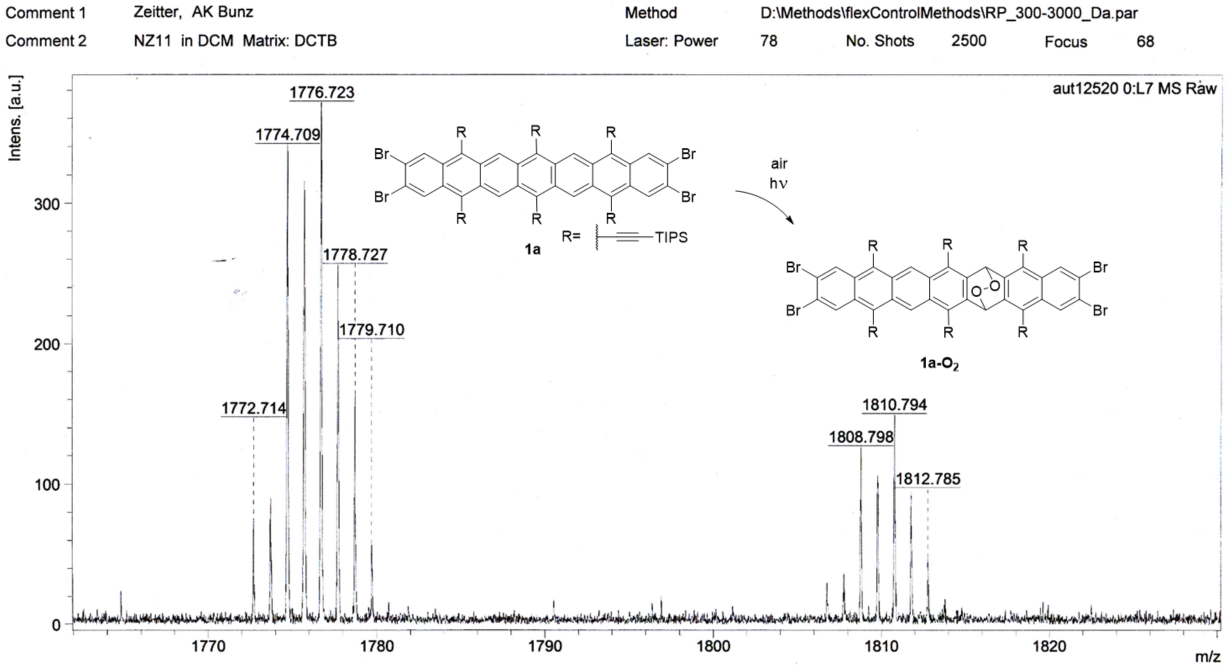

Figure S38. MALDI-MS (DCTB, pos. mode) of the product obtained from degradation under ambient conditions showing oxo-adduct **1a-O$_2$**.

2.7 OFET Fabrication and Characterization

A sliced, highly doped silicon wafer with 100 nm thick thermally grown SiO_2 was successively cleaned via ultra-sonication in acetone, isopropanol and ethanol, each for 10 min. It was washed with water and dried in a stream of nitrogen. The wafer was placed in freshly produced *Caro´s* acid and was heated to 100 °C for 20 min. After cleaning with water and drying, a 150 mM solution of $Al(NO_3)_3$ * 9 H_2O in ethanol was spin-coated (5000 rpm; 40 s) onto the substrate. Right after that the wafer was heated to 300 °C for 30 min. For the formation of the self-assembled monolayer, the substrate was placed in a 15.0 mM solution of 12-cyclohexyldodecylphosphonic acid (CDPA)[10] in isopropanol at room temperature for 16 h. Then the substrate was cleaned via ultra-sonication in isopropanol for 10 min, rinsed with water and dried in a stream of nitrogen. The capacitance of the dielectric layer amounted to 26 nF cm^{-2}.

Drop-cast thin-films were prepared by dropping the prepared solution (**1a**: 1.50 mg/mL in toluene; **1c**: 0.75 mg/mL toluene) onto the heated substrate (50 °C) covering the wafer. Electrode formation was achieved by depositing a 40 nm thick layer of gold was deposited through a shadow mask onto the organic layer in a vacuum evaporator at a pressure below 2 x 10^{-6} bar. Transistor characteristics were measured with a semiconductor characterization system (Keithley 4200-SCS) in a nitrogen filled glove box. The field effect mobilities were determined in the saturated regime using the equation $I_{DS} = (\frac{W}{2L})C_i\mu(V_G - V_{th})^2$, where I_{DS} is the source-drain current, W is the channel width, L the channel length, C_i is the capacitance per unit area of the gate dielectric layer, μ is the field effect mobility, and V_{th} is the threshold voltage.

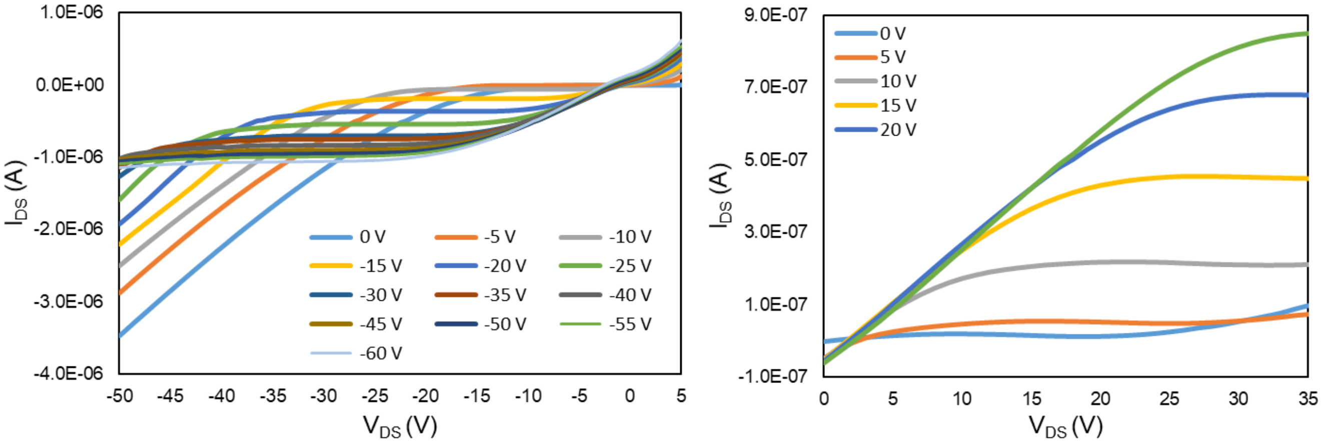

Figure S39. OFET output characteristics of heptacene **1a;**

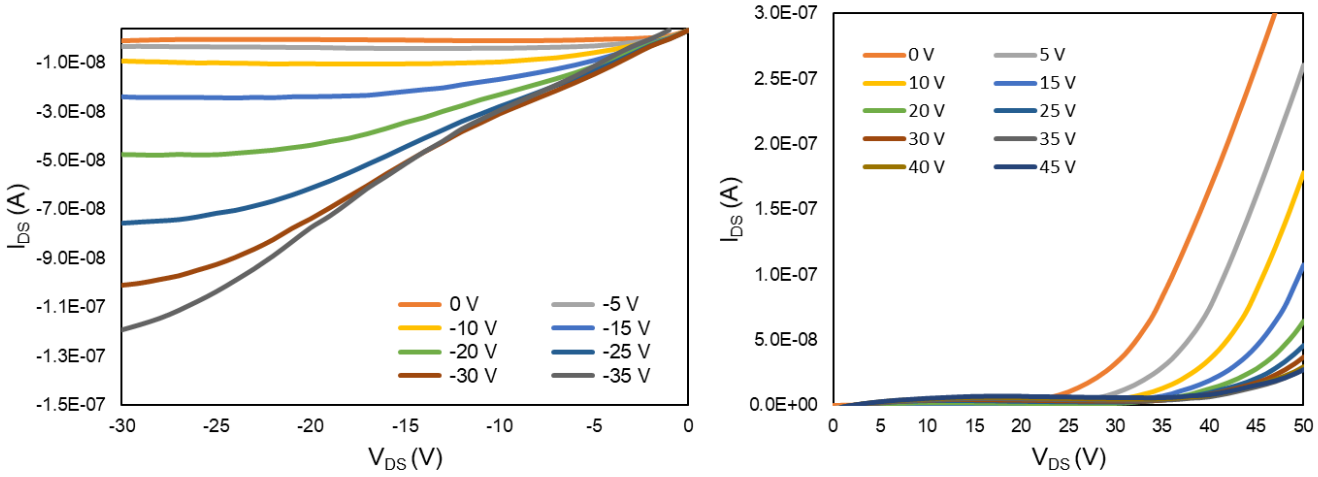

Figure S40. OFET output characteristics of heptacene **1c**

2.8 X-ray diffraction (XRD)

XRD characterization of thin-films of the heptacenes **1a** and **1c** on the CDPA modiefied substrates were carried out on a Rigaku Smartlab X-Ray Diffractometer with assignment to the (hkl) values. The data shows:

- X-ray diffraction peaks of the film of **1a** correspond to the (h00) diffraction derived from the single crystal with interlayer spacing $d_{crystall}$ (100) = 19.6 Å. (d_{film} (h00) = 19.7 Å).
- X-ray diffraction peaks of the film of **1c** correspond to the (00l) diffraction derived from the single crystal with $d_{crystall}$ (001) = 37.9 Å. (d_{film} (00l) = 37.7 Å).

Corresponding lattice planes are parallel to the substrate surface, thus the π planes are perpendicular to the surface (**1a**: 88.4°, **1c**: 89.7°).

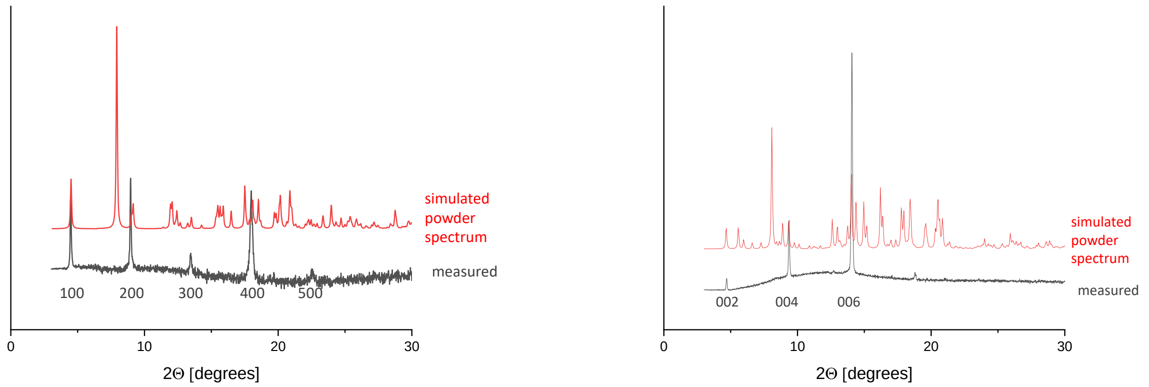

Figure S41: X-ray diffraction of thin films of **1a** (left) and **1c** (right)

2.9 EPR Spectroscopy

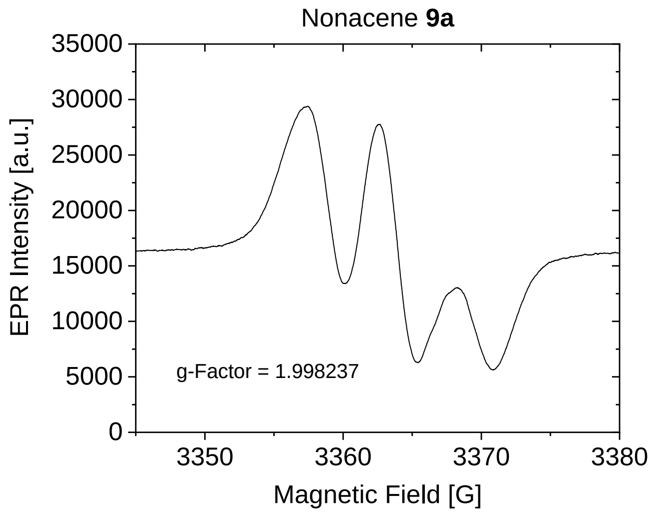

Figure S42: EPR spectrum of **9a** in DCM (9.42 GHz, RT).

2.10 Oxidation and Reduction Experiments

Radical anion **1a**$^{\cdot-}$: Heptacene **1a** (15.0 mg, 8.44 µmol, 1.00 eq.) was dissolved in 3 mL anhydrous THF under inert atmosphere. A freshly prepared solution (0.529 mL, 0.160 M) of [K(18-crown-6)(THF)$_2$] anthracenide (4.07 mg, 8.44 µmol, 1.00 eq) was added and the mixture was stirred for 30 min. The reaction progress was controlled by UV/vis spectroscopy and EPR spectroscopy. Dark crystals were formed by slow evaporation of the solvent at RT.

Radical cation **1a**$^{\cdot+}$: To a stirring solution of Heptacene **1a** (25.0 mg, 38.8 µmol, 1.00 eq.) in 4 mL DCM, $AgSbF_6$ (13.32 mg, 38.8 µmol, 1.00 eq.) in 0.5 mL acetonitrile was added dropwise. After 30 min no change in the UV/vis spectrum was observed. The reaction mixture was stirred for 12 h at RT and changed colour from brown to red. After that, the mixture was filtered, and the solvent was slowly evaporated. The formation of the radical cation **1a**$^{\cdot+}$ was observed via UV/vis- and EPR-spectroscopy. Unfortunately no single crystals were obtained.

Further reduction experiments:

In addition, an attempt was made to generate a dianion of heptacene **1a**. To a stirred solution of heptacene **1a** (15.0 mg, 8.44 µmol, 1.00 eq.) in 3 mL anhydrous THF under inert atmosphere an excess of 0.10 mL sodium potassium alloy (Sodium 22 wt. %, Potassium 78 wt. %) was added and the solution was stirred for 30 min at RT. The reaction progress was controlled by UV/vis spectroscopy and EPR spectroscopy. After 30 min stirring the absorption (λ_{max}) of heptacene **1a** disappeared and a weak absorption at 1400 nm was observed. The EPR spectrum of the reaction mixture also showed a weak signal. The UV/VIS spectrum and the EPR spectrum do not indicate the formation of a dianion of heptacene **1a**.

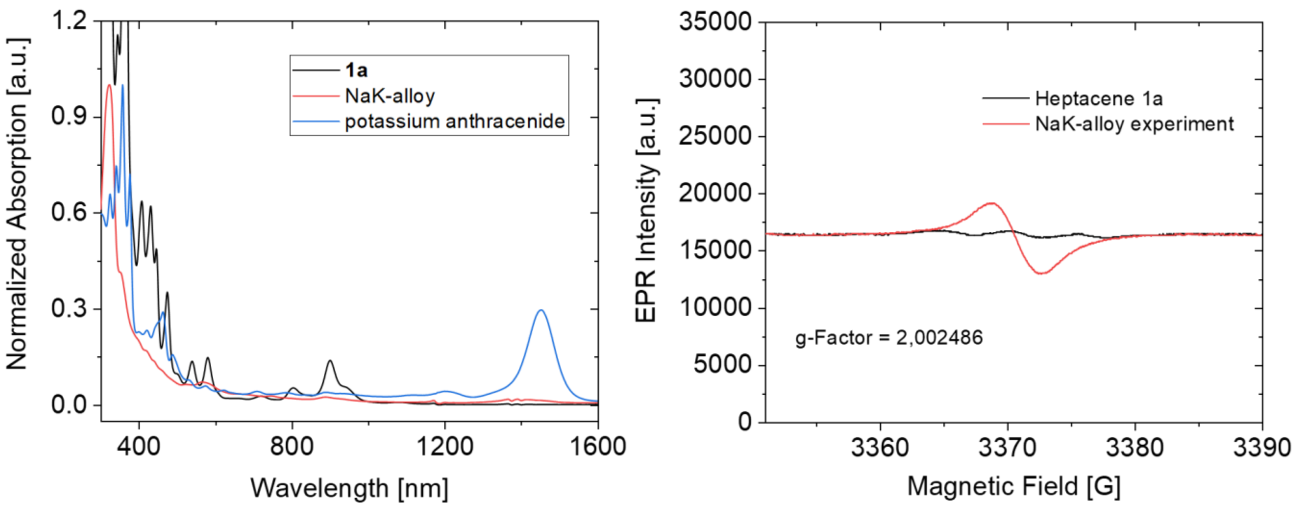

Figure S43: Left: Normalized UV/vis absorption spectra of the product of the reduction with NaK alloy (red), the reduction with potassium anthracenide (blue) and heptacene **1a** (black) in anhydrous THF at RT under inert atmosphere. Right: EPR spectrum of **1a** (black) and the reaction product of **1a** and sodium potassium alloy in anhydrous THF under inert atmosphere (9.45 GHz, RT)

3 References

[1] G. Sheldrick, *Acta Cryst. A* **2015**, *71*, 3-8.
[2] G. Sheldrick, *Acta Cryst. C* **2015**, *71*, 3-8.
[3] G. R. Fulmer, A. J. M. Miller, N. H. Sherden, H. E. Gottlieb, A. Nudelman, B. M. Stoltz, J. E. Bercaw, K. I. Goldberg, *Organometallics* **2010**, *29*, 2176-2179.
[4] M. J. F. Gaussian 16, G. W. Trucks, H. B. Schlegel, G. E. Scuseria, M. A. Robb, J. R. Cheeseman, G. Scalmani, V., G. A. P. Barone, H. Nakatsuji, X. Li, M. Caricato, A. V. Marenich, J. Bloino, B. G. Janesko, R. Gomperts, B. Mennucci, H. P. Hratchian,, A. F. I. J. V. Ortiz, J. L. Sonnenberg, D. Williams-Young, F. Ding, F. Lipparini, F. Egidi, J. Goings, B. Peng, A. Petrone, T. Henderson,, V. G. Z. D. Ranasinghe, J. Gao, N. Rega, G. Zheng, W. Liang, M. Hada, M. Ehara, K. Toyota, R. Fukuda, J. Hasegawa, M. Ishida, T., Y. H. Nakajima, O. Kitao, H. Nakai, T. Vreven, K. Throssell, J. A. Montgomery, Jr., J. E. Peralta, F. Ogliaro, M. J. Bearpark, J. J. Heyd, E., K. N. K. N. Brothers, V. N. Staroverov, T. A. Keith, R. Kobayashi, J. Normand, K. Raghavachari, A. P. Rendell, J. C. Burant, S. S. Iyengar,, M. C. J. Tomasi, J. M. Millam, M. Klene, C. Adamo, R. Cammi, J. W. Ochterski, R. L. Martin, K. Morokuma, O. Farkas, J. B. Foresman, and, G. D. J. Fox, Inc., Wallingford CT,, **2016**.
[5] Y. Shao, L. F. Molnar, Y. Jung, J. Kussmann, C. Ochsenfeld, S. T. Brown, A. T. B. Gilbert, L. V. Slipchenko, S. V. Levchenko, D. P. O'Neill, R. A. DiStasio Jr, R. C. Lochan, T. Wang, G. J. O. Beran, N. A. Besley, J. M. Herbert, C. Yeh Lin, T. Van Voorhis, S. Hung Chien, A. Sodt, R. P. Steele, V. A. Rassolov, P. E. Maslen, P. P. Korambath, R. D. Adamson, B. Austin, J. Baker, E. F. C. Byrd, H. Dachsel, R. J. Doerksen, A. Dreuw, B. D. Dunietz, A. D. Dutoi, T. R. Furlani, S. R. Gwaltney, A. Heyden, S. Hirata, C.-P. Hsu, G. Kedziora, R. Z. Khalliulin, P. Klunzinger, A. M. Lee, M. S. Lee, W. Liang, I. Lotan, N. Nair, B. Peters, E. I. Proynov, P. A. Pieniazek, Y. Min Rhee, J. Ritchie, E. Rosta, C. David Sherrill, A. C. Simmonett, J. E. Subotnik, H. Lee Woodcock Iii, W. Zhang, A. T. Bell, A. K. Chakraborty, D. M. Chipman, F. J. Keil, A. Warshel, W. J. Hehre, H. F. Schaefer Iii, J. Kong, A. I. Krylov, P. M. W. Gill, M. Head-Gordon, *Phys. Chem. Chem. Phys.* **2006**, *8*, 3172-3191.
[6] B. Purushothaman, M. Bruzek, S. R. Parkin, A.-F. Miller, J. E. Anthony, *Angew. Chem., Int. Ed.* **2011**, *50*, 7013-7017.
[7] M. Müller, PhD thesis, University of Heidelberg (Heidelberg), **2019**.
[8] D. Xia, X. Guo, L. Chen, M. Baumgarten, A. Keerthi, K. Müllen, *Angew. Chem., Int. Ed.* **2016**, *55*, 941-944.
[9] M. Müller, S. Maier, O. Tverskoy, F. Rominger, J. Freudenberg, U. H. Bunz, *Angew. Chem., Int. Ed.* **2020**, *59*, 1966.
[10] X. Xu, Y. Yao, B. Shan, X. Gu, D. Liu, J. Liu, J. Xu, N. Zhao, W. Hu, Q. Miao, *Advanced Materials* **2016**, *28*, 5276-5283.

4 Author Contributions

Nico Zeitter: Synthesis, analysis, stability studies, cyclic voltammetry measurements, writing of original draft (lead)
Nikolai Hippchen: Device fabrication and characterization (supporting)
Steffen Maier: editorial work (supporting)
Andreas Dreuw: editorial work (supporting)
Frank Rominger: Crystal structure elucidation (supporting)
Jan Freudenberg: Project administration, editorial work (supporting)
Uwe H. F. Bunz: Writing of original draft, project administration, funding acquisition (lead)
